# Punishment is slower than cooperation or defection in online network games

**DOI:** 10.1038/s41598-024-72939-2

**Published:** 2024-10-03

**Authors:** George Dewey, Hiroyasu Ando, Ryo Ikesu, Timothy F. Brewer, Ryunosuke Goto, Akihiro Nishi

**Affiliations:** 1 Department of Epidemiology, UCLA Fielding School of Public Health, Los Angeles, CA 90095 USA; 2 Present Address: Machine Intelligence Group for the Betterment of Health and the Environment, Network Science Institute, Boston, MA 02115 USA; 3 Department of Biostatistics, UCLA Fielding School of Public Health, Los Angeles, CA 90095 USA; 4 Division of General Internal Medicine and Health Services Research, David Geffen School of Medicine at UCLA, Los Angeles, CA 90024 USA; 5 Division of Infectious Diseases, David Geffen School of Medicine at UCLA, Los Angeles, CA 90024 USA; 6 Department of Pediatrics, The University of Tokyo Hospital, Bunkyo, Tokyo, Japan; 7 California Center for Population Research, University of California Los Angeles, Los Angeles, CA 90095 USA; 8 Bedari Kindness Institute, University of California Los Angeles, Los Angeles, CA 90095 USA

**Keywords:** Evolution of cooperation, Punishment, Decision time, Time pressure, Social evolution, Dynamic networks

## Abstract

Punishment serves as a balancing force that dissuades people from acting selfishly, which complements cooperation as an essential characteristic for the prosperity of human societies. Past studies using economic games with two options (cooperation and defection) reported that cooperation decisions are generally faster than defection decisions and that time pressure possibly induces human players to be more intuitive and thus cooperative. However, it is unclear where punishment decisions sit on this time spectrum. Therefore, we recruited human players and implemented two series of online network games with cooperation, defection, and punishment options. First, we find that punishment decisions are slower than cooperation or defection decisions across both game series. Second, we find that imposing experimental time pressure on in-game decisions neither reduces nor increases the frequency of punishment decisions, suggesting that time pressure may not directly interact with the mechanisms that drive players to choose to punish.

## Introduction

Punishment is widely observed in various forms: recent and ongoing examples include the gun violence epidemic in the United States^1,2^, the waging of war under the guise of security or using punishment as a just cause for war^3–6^, institutional responses to crimes^7^, and anti-prevention behavior during the COVID-19 pandemic^8–10^. Games are no exception to this rule; for example, in competitive, multiplayer games, players may choose to sacrifice some of their own chances of victory to substantially reduce the chances of victory for their opponents. In all these scenarios, punishment involves *paying a cost to harm others* and serves as an important counterpart to cooperation (paying a cost to benefit others), a characteristic that has been integral to the formation of human societies^11–16^. However, in addition to the costs associated with choosing to punish, punishers incur societal and emotional penalties: institutions that punish in unjust or cruel ways are labeled tyrannical^17^, while individuals who punish others make themselves a target for retaliation or reduced reputation^18,19^. This divide between punishment’s potentially important relationship with cooperation and the negatives associated with choosing to punish contributes to a lack of clarity about the mechanisms that drive punishment. Researchers have generally proposed that punishment serves to sustain cooperation through negative reinforcement: prominent theories suggest that punishment promotes the development of cooperation in social networks by reducing the payoff of defectors and converting them into cooperators^20–22^ or that punishment acts as a balancing mechanism that allows people to reduce perceived inequality by allowing poorer individuals to spend a small amount to take a larger amount away from their richer neighbors^23–27^.

To better understand the dynamic between cooperative and non-cooperative behavior, researchers formulated the social heuristics hypothesis (SHH), which applies a dual process perspective to people’s decision-making. Under the SHH, people develop intuitive routines which they can apply to social scenarios through daily interactions with others; this intuition then competes with deliberation when people are spurred to make decisions. However, studies that evaluate the SHH^28–30^ have generally employed economic games where players could only choose to cooperate or defect (paying no cost and not affecting others) with each other. Follow-up studies using the same two-option setup introduced using decision times in experimental games to better understand the dynamics between cooperation and defection^28,31–34^. One such study found that choosing cooperation is faster than choosing defection when participants have many cooperative neighbors but slower when one’s neighbors are less cooperative^32^. This finding may be the result of “decision conflict”^35^: a mismatch between the current state of connected neighbors (the “social environment”) and the participant’s intended choice, eliciting a feeling of conflict, which slows down decision-making speed; this conflict may compound the general sense that defection may provoke more feelings of conflict than cooperation. Other studies^28,31^ found that participants’ intuition and social heuristics may prefer cooperation over defection, and therefore, when time pressure (limiting the amount of time available for participants to make decisions) was imposed, participants might be more likely to mobilize intuition over deliberation, resulting in more frequent cooperation. However, these findings have not been clearly reproduced in follow-up studies^36–38^. We aimed to build upon the results of these studies evaluating the SHH, decision conflict, and decision times, by introducing the punishment option to an experimental framework which allowed players in a network environment to interact with each other. In doing so, we present players in our games with a series of complex decision points in which they could not only contribute to the benefit of others (by cooperating) but also detract from other players (by punishing) in the same game.

In this study, we examine the unexplored relationship between punishment and decision time using two experiments consisting of online network games to better understand how punishment decision-making differs from decision-making that leads to cooperation or defection. In Experiment 1, we compared the decision times of punishment to those of cooperation and defection. Here, we hypothesized that decisions involving punishment would take longer than those involving cooperation or defection in general (regardless of decision mismatches) because choosing to punish requires players to decide to pay an immediate cost, give up any potential fitness gains from reciprocal cooperation in the short term, and prepare for potential retaliation from those who are punished. Then, in Experiment 2, we determined if experimental time pressure could alter the distribution of players’ decisions by reducing the occurrence of punishment and increase the occurrence of cooperation. By minimizing the amount of time available to make decisions, we would expect players to be inclined to make “intuitive” choices – that is, to opt to cooperate and not to punish.

## Methods overview

### Experimental design

We implemented two series of repeated public goods game (PGG) by adapting an online, network-based framework^32,39^ previously used to study cooperation and decision time by introducing a punishment option. The games allow players to interact with a dynamic group of other players over time and strategize ways to improve their status based on the decisions and statuses of others. Players were recruited from around the world using Amazon Mechanical Turk (MTurk) between March 2018 and December 2018 for Experiment 1 and between March 2023 and May 2023 for Experiment 2. The experiments were approved by and performed according to guidelines and regulations set by the UCLA Office of Research Administration (UCLA IRB#16-001920). Informed consent was obtained online from all participants. At the end of all game sessions, accumulated in-game wealth was converted at a rate of 2000 points to 1 USD; players were also compensated USD 3 for participating in an experimental game session. Recruited players were assigned to dynamic networks composing a single game session and asked to interact in the PGG over 15 rounds. Each network was generated by arranging each game session’s players into a Erdős-Renyi random graph in which 30% of all possible ties were present at the start of the experiment.

**Fig. 1:**
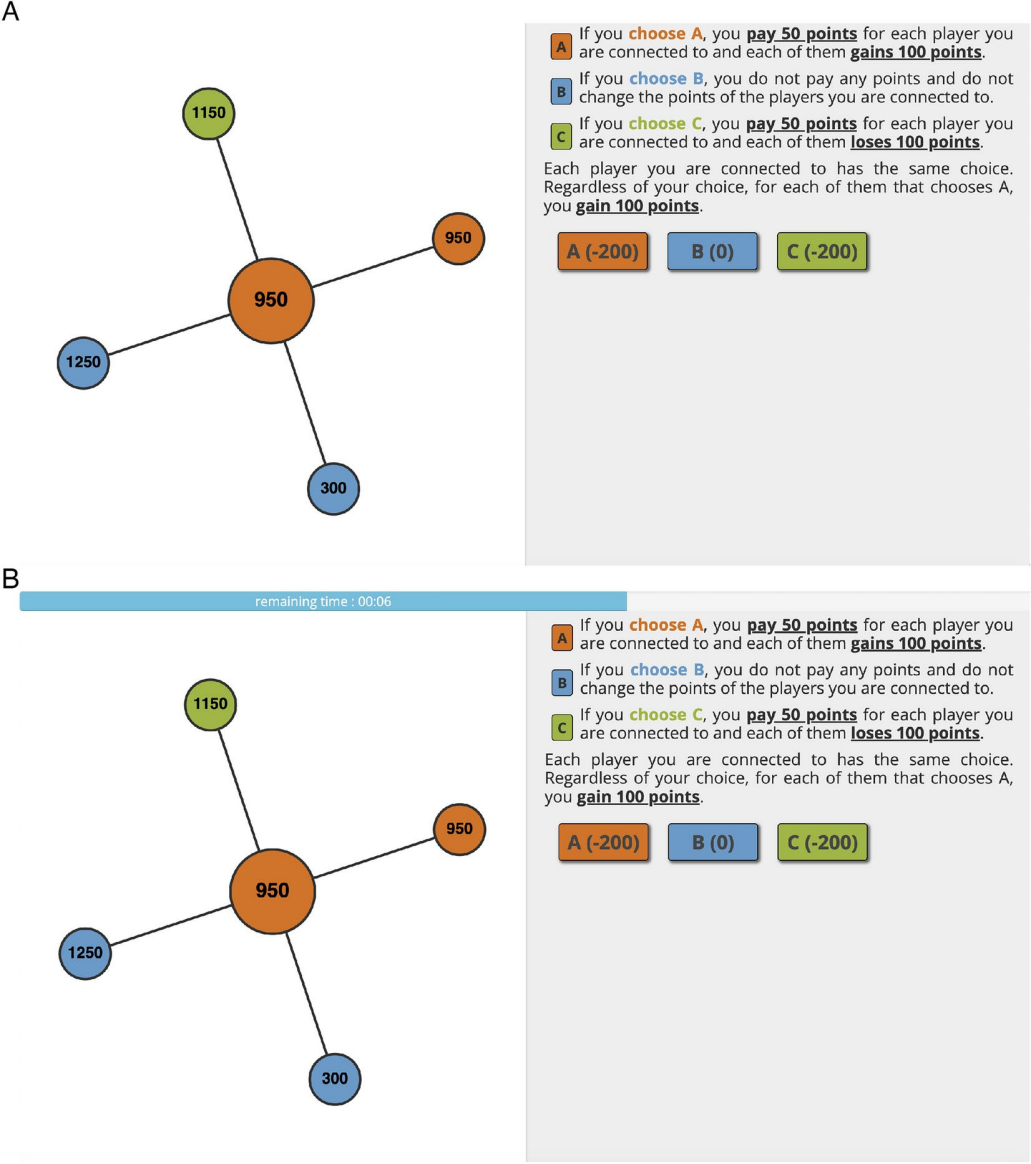
Example player’s screenshot without (**A**) and with (**B**) time pressure. The focal player is represented by the larger, central circle highlighted in orange, while the smaller surrounding circles represent connected players in the same game session (the colors of the circles indicate the choice of each player in the round prior). In the setting with time pressure (**B**), a horizontal bar appeared on the player’s screen showing the remaining time the player had to make their decision (this setting only appeared in Experiment 2). In the setting without time pressure (**A**), no time pressure was implemented, and no bar appeared; panel A is representative of all sessions in Experiment 1. Values in the circles represent players’ in-game points.

Players were first invited to play two practice rounds to introduce them to the game session format and the layout of elements within the experimental platform (Fig. 1). Once these two practice rounds were completed, all players in the game session were randomly allocated a high or low quantity of in-game points and asked to interact with each other. Game sessions that did not recruit enough players from MTurk did not progress to the practice round stage and were closed to further entry; players who previously completed a game session were prohibited from participating in future game sessions.

At the start of each game session round, players entered the decision phase, where they were given one of three options: cooperation (the player pays 50 points per connected player to have all connected players gain 100 points), defection (the player pays nothing, not affecting other players), and punishment (the player pays 50 points per connected player to have all connected players lose 100 points). No institutional/central punishment^40^ or sanctioning^41^ occurred. Each option was indiscriminate: players could not choose to interact with some connected players and not interact with others and the option that was selected resulted in the appropriate change to all connected players. Once players made their decisions, they were shown the decisions made by other connected players in the decision phase and an updated tally of in-game points for the focal player and any connected players was shown. In Experiment 1, game sessions were randomly assigned to have neighboring players’ wealth be visible or invisible (with the update at the end of the decision phase mirroring this randomization); however, statistical analysis (not shown) implied that there was no effect of wealth visibility on the frequency of punishment or decision times, allowing us to keep wealth visible in all sessions of Experiment 2. The effect of wealth visibility on other outcomes such as subjective well-being has been reported elsewhere (without examining or including decision time in analyses)^42^. In the same vein, we found no association between decision times and initial point allocation; we therefore excluded point allocation from the main analyses presented in this study.

Players were then allowed to update their network connections by answering a series of questions in the rewiring phase. In this phase, 30% of all possible ties in the network were randomly selected by *breadboard*. For each extant tie, one player from the tie pairing was chosen at random and asked if they wanted to break the tie. If the player chose to break the tie, the connection was broken for the next round; if the player chose not to break the tie, the tie was retained. For each non-extant tie, both players in the potential tie were asked if they wanted to make a new connection; if and only if both players agreed, the tie was constructed and carried over to the next game round. The networks were then reconstructed based on the results of the rewiring phase and the subsequent round began until all 15 rounds of the game were completed.

### Decision time

For this study, we defined decision time (a.k.a. response time) of the cooperation-defection-punishment decision-making as the time interval between two timestamps which were recorded by *breadboard*: the first timestamp recorded the appearance of the screen which allowed players to choose between cooperation, defection, or punishment (shown in Fig. 1), while the second timestamp recorded when players clicked one of the buttons representing cooperation, defection, or punishment.

### Punishment mechanisms

To guide our analysis of the different rationales for punishment decisions in our games, we classified punishment decisions into four categories based on prevailing theorized punishment mechanisms in the literature^20,25–27,43^. These mechanisms are not mutually exclusive and can overlap.

First, we classified punishment for *copying or retaliation* as punishment that occurred when at least one connected player in the prior round chose to punish. Punishment of this type may be beneficial because copying others’ behavior and learning from copying is known to be advantageous regardless of the reason for the copied behavior^44,45^. Retaliation occurs when the punisher chooses to punish punishers from the last encounter to dissuade them from punishing in the future^43,46–48^. Revenge is a related concept which pairs retaliation with an emotional argument that punishers should be punished in return. Unfortunately, our experimental setting cannot precisely distinguish between these related mechanisms and therefore we combine punishment decisions that could be classified as supported by either mechanism into one single category.

Second, we defined punishment for *negative reinforcement* as punishment that occurred when the cooperation rate among connected players in the prior round was less than 50%. In our experimental setting, players paid 50 units for each connecting player when choosing to cooperate. As a result, the cost–benefit ratio of choosing cooperation was 0.5 (as the cost of cooperation is greater than the expected benefits since connected players could choose either to defect or punish); this means that cooperation would decay very quickly if the cooperation rate fell below 50%. Therefore, punishing connected neighbors may contribute to long-term reciprocal cooperation in social networks when the networks are not very cooperative^20,21,49,50^.

Third, we defined punishment for *inequality aversion* as punishment that occurred when a focal player’s wealth was less than the average wealth of connected players in the prior round. Since the cost to punish is on average lower than the expected loss from being punished, the degree of economic inequality (the difference of in-game points) is reduced when poorer players punish connected richer players. Therefore, players could be motivated to punish by inequality aversion^25–27,51^.

Fourth and finally, punishment decisions that did not meet any of the above three definitions were classified as unclassified or de novo punishment, possibly reflecting a base inclination to choose punishment. It is possible that some players wanted to “try out” the punishment option even if they were not poor and connected to mainly cooperative neighbors.

### Statistical analysis

The structure of the game sessions and the arrangement of players into networks meant that multiple observations were made for a single player across multiple rounds in each session. We account for this hierarchical data structure using multilevel random intercepts models, utilizing R version 4.3.1^52^ and the *lme4* statistical package. Detailed results from these multilevel models are listed in the Supplementary Materials.

While prior work^28,32,53^ utilized a log_10_-transformation when analyzing decision times because decision times are generally only left-bounded by zero, time data from games involving time pressure would also be right-bounded by the time limit. As a result, we chose to omit the transformation to keep model estimates from limited and non-limited data on the same scale.

## Experiment 1

### Results

**Fig. 2:**
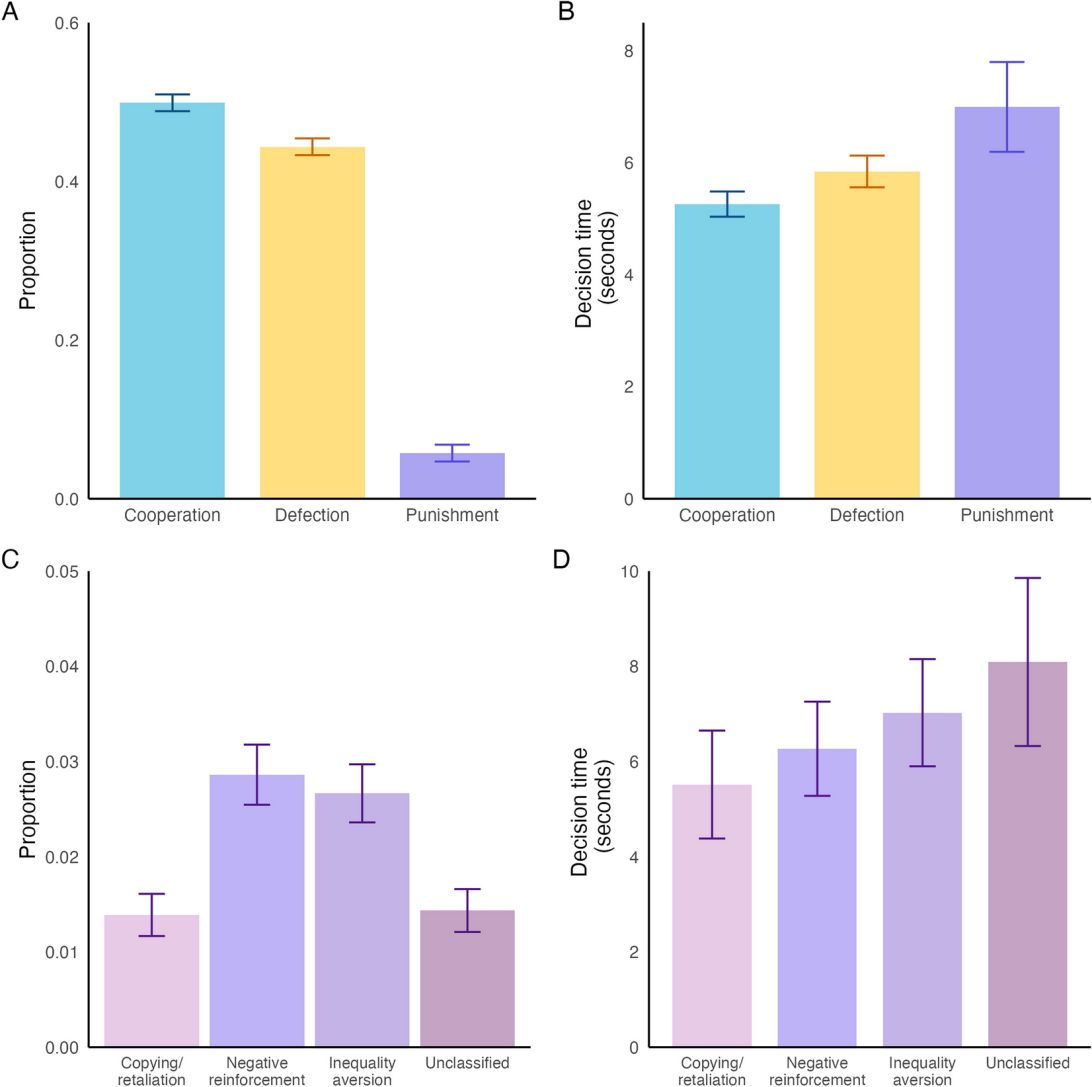
Punishment is rare and slower than cooperation or defection in Experiment 1. (**A**) Cooperation and defection are more common. Punishment is relatively uncommon (6.6%). (**B**) On average, punishment decisions took longer to make compared to defection or cooperation. (**C**) The proportion of decisions reflecting each of the mechanisms of punishment. The total proportion represented in panel C exceeds the actual proportion of punishment decisions in the experiment because the mechanism categories can overlap. (**D**) Mean decision times for each punishment mechanism. The mechanisms display a tendency to slow down as the complexity of the punishment decision increases. Bars indicate 95% confidence intervals of proportions or decision time.

In Experiment 1, 719 unique players (mean: 14.9/game, range: 9–25/game) made 9,776 decisions of cooperation, defection, or punishment. Cooperation was chosen 4878 times (49.4%, 95% confidence interval for proportion [CI] 48.4–50.5%), defection was chosen 4,336 times (43.9%, 95% CI 42.9–45.0%), and punishment was chosen 562 times (6.6%, 95% CI 5.6–7.7%) **(**Fig. 2A**)**. The mean degree (the number of connections a player had at any moment) in Experiment 1 was 5.9 (range: 0–17). At the end of 15 rounds, the mean accumulated wealth across all players was 1584 in-game points (equivalent to USD 0.79). Therefore, combined with the USD 3 participation award, players in Experiment 1 obtained USD 3.79 on average.

We found that that punishment decisions (mean = 7.0 s, 95% CI 6.2–7.8 s) are slower than cooperation decisions (mean = 5.3 s, 95% CI 5.0–5.5 s, *p* of punishment vs. cooperation from random intercepts model: 0.004) and defection decisions (mean = 5.8 s, 95% CI 5.6–6.1 s, p of punishment vs. defection: 0.032) (Fig. 2B; Supplementary Table S1).

Punishment due to copying or retaliation encompassed 1.4% (149 instances) of all decisions in Experiment 1 (Fig. 2C) and the mean decision time of such decisions was 5.5 s (95% CI 4.4–6.7 s) (Fig. 2D), which tended to be the fastest among the four punishment categories (p vs. negative reinforcement = 0.66, p vs. inequality aversion = 0.16, p vs. unknown punishment = 0.02, p vs. all three other categories = 0.01). Punishment due to negative reinforcement comprised 2.9% (307 instances) of all decisions (Fig. 2C) with a mean decision time of 6.3 s (95% CI 5.3–7.3 s) (Fig. 2D), while punishment due to inequality aversion included 2.7% (286 instances) of all decisions (Fig. 2C) with a mean decision time of 7.0 s (95% CI 5.9–8.2 s) (Fig. 2D). Unclassified punishment encompassed 1.4% (154 instances) of all decisions (Fig. 2C) with a mean decision time of 8.1 s (95% CI 6.3–9.9 s) (Fig. 2D); these decisions tended to be longer than punishment for inequality aversion (p = 0.73) and longer than punishment decision times for the combination of the three abovementioned mechanisms (p = 0.04). Furthermore, having a punisher among one’s network neighbors in the previous round (that is, having been punished in the previous round) was associated with a 2.4 s decrease in punishment decision time (p = 0.009, Supplementary Table S5).

We also conducted several robustness checks to verify our findings. We evaluated the potential interaction between choosing to punish and the rate of punishment among connected players in the prior round; multilevel models including an interaction term (Supplementary Table S6) did not identify any substantial evidence supporting such an interaction. We also verified that the different levels of initial point allocation in Experiment 1 did not influence the distribution of decisions or decision times as discussed previously (Supplementary Tables S7, S8). Additionally, we considered if there was potential for the wealth visibility condition described in the Methods Overview to specifically influence inequality aversion-related punishment. Supplemental analyses (Supplementary Tables S9, S10, S11) indicated that there was no association between wealth visibility and the frequency or decision times of inequality aversion punishment decisions, suggesting that future experiments could proceed without additional consideration for wealth visibility status.

### Discussion

Experiment 1 allowed us to determine how decision times associated with different mechanisms of punishment relate to one another to better understand why punishment was on average slower than either cooperation or defection. The relative quickness of punishment for copying or retaliation compared to the other punishment mechanisms may reflect players needing less time to justify choosing to punish if connected players have punished previously, especially since punishment was relatively infrequent compared to cooperation and defection and therefore players were not frequently connected to others who had punished before. Punishment for negative reinforcement was slower compared to punishment for copying or retaliation; this difference may be a product of players needing additional time to choose to punish to stop a perceived decay in cooperation in their social network. Increased decision time associated with inequality aversion is also understandable since punishing richer neighbors is a valid in-game strategy which takes a more self-interested viewpoint; players choosing this strategy may need more time to contemplate about their economic position in relation to other players in the session as opposed to the overall wealth of the group (which would apply more to the two previously mentioned mechanisms for punishment). We speculate that unclassified punishment having the longest average decision time may reflect the degree of feelings of decision conflict and may be triggered by feelings of remorse for taking points away from other players.

In summary, the small differences in decision time over the four punishment categories did not give us conclusive evidence about the specific mechanisms that slow down the punishment decision-making process. However, each of the mechanisms we assessed relate to a conflict that players can aim to resolve using the “negative” punishment option. For example, in copying or retaliation punishment, some proportion of connecting players are no longer cooperative and thus may deserve to be punished. Because our experimental setting requires players to make a decision that will affect all connected players, choosing to punish to copy or as retaliation will require players to punish both punishers and cooperators; such a situation could elicit feelings of conflict.

## Experiment 2

Since Experiment 1 showed that punishment decisions are slower than cooperation or defection decisions, we hypothesized that setting a time limit on decision-making could reduce the number of punishment decisions (Hypothesis 1). However, we also considered an alternative hypothesis: since most of the punishment decisions that occurred in Experiment 1 were linked to postulated mechanisms, the number of punishment decisions would be unchanged unless the context that led to the punishment decision was addressed (Hypothesis 2).

To reconcile these two hypotheses, we developed Experiment 2, a second experimental series of 50 games which were randomly divided into 25 games with a time pressure setting (the TP + setting) (Fig. 1B), in which player decisions had to be made in 3 s or less, and 25 other games with no time pressure (the TP- setting) (Fig. 1A). In the TP + setting, if players did not confirm their choice within the time limit, the system automatically repeated their decision from the previous round; if players missed two decision-making opportunities due to inactivity, they were dropped from the remainder of the session (Supplementary Fig. S1). We chose to implement time pressure in Experiment 2 as a three-second limit on decision-making; we based our selection of three seconds on the distribution of decision times in Experiment 1 (Supplementary Fig. S2). All players in networks assigned the TP + condition had time pressure imposed for every in-game round. Players in networks assigned the TP- condition played a game identical to the one played in Experiment 1.

### Results

In Experiment 2 (across both TP + and TP- settings), 739 players (mean: 14.8/game, range: 8–20/game) made 10,654 decisions. Cooperation was chosen 4,185 times (39.3%, 95% CI: 38.3–40.3%), defection was chosen 5,790 times (54.4%, 95% CI: 53.4–55.4%), and punishment was chosen 679 times (6.4%, 95% CI: 5.4–7.4%). The mean degree across sessions in Experiment 2 was 5.7 (range: 0–16). At the end of 15 rounds, the mean accumulated wealth was 935 in-game units (equivalent to USD 0.47); with the USD 3 participation award, the average reward for participants in Experiment 2 was USD 3.47.

**Fig. 3:**
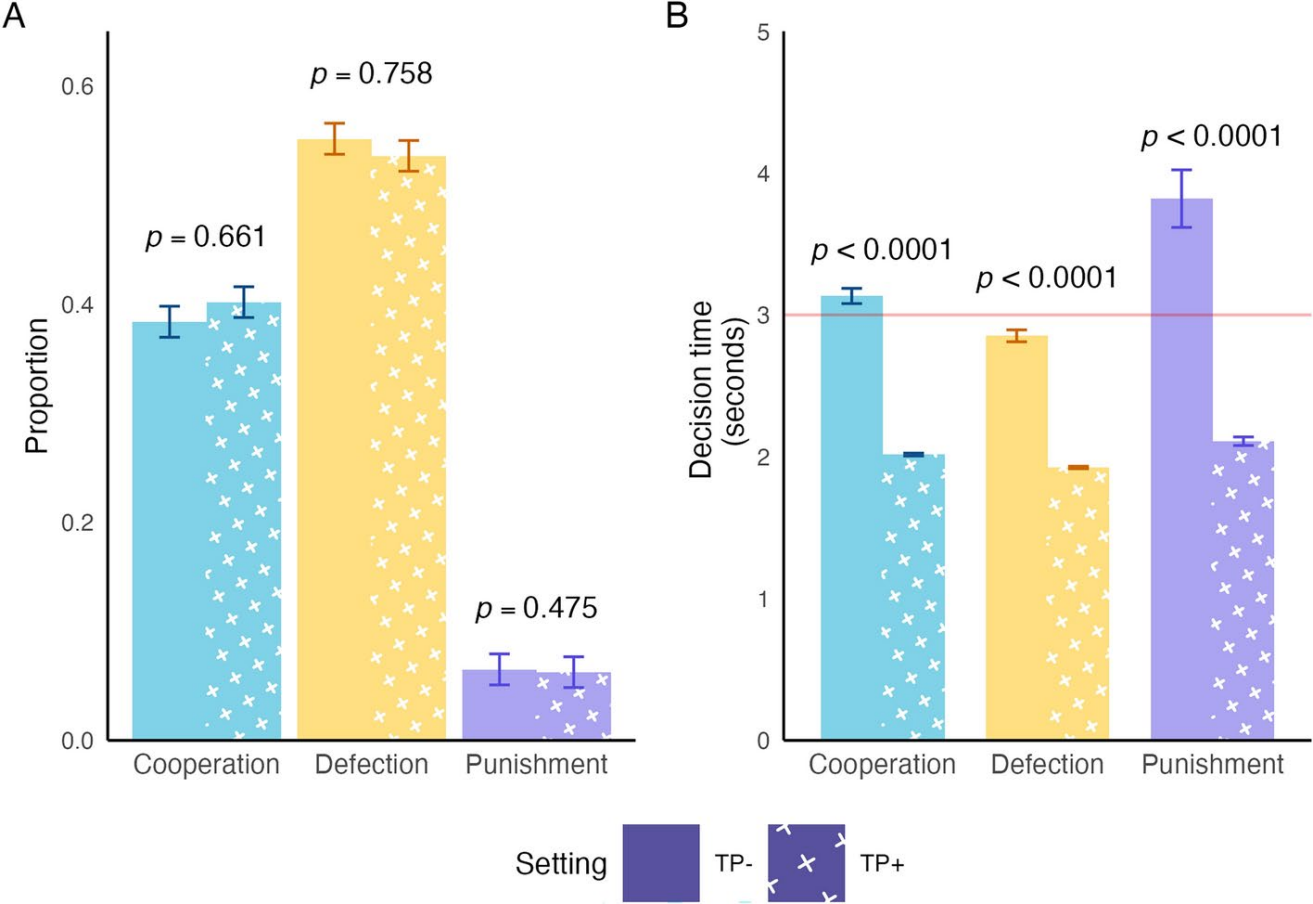
Punishment is slower than cooperation or defection, even under time pressure. (**A**) There was no substantial difference in the frequencies of each decision type comparing sessions with time pressure (TP-; left, bars without crosses) and sessions with time pressure (TP + ; right, bars with white crosses). (**B**) Punishment was slower than cooperation and defection in both the TP- and TP + settings. The red line indicates the three-second time pressure boundary. Error bars represent 95% confidence intervals of proportions or means.

In the TP + setting, punishment was chosen 338 times out of 5,407 decisions (6.3%, 95% CI 4.8–7.7%), while in the TP- setting, punishment was chosen 341 out of 5,247 decisions (6.5%, 95% CI 5.1–7.9%). While there was no substantial difference in the frequency of punishment comparing the TP + and TP- setting (p = 0.475) (Fig. 3A), all decision-making was faster in the TP + games as expected (Fig. 3B), suggesting that Experiment 2 was successfully implemented.

**Fig. 4:**
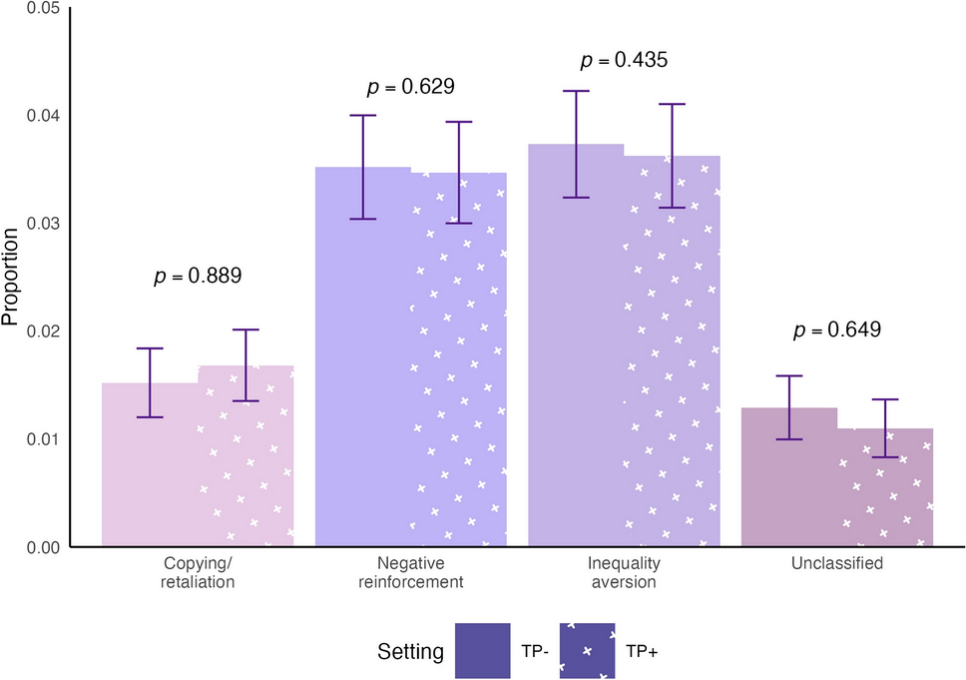
Time pressure does not change the frequency of mechanism-specific punishment. In Experiment 2, we observed no substantial difference in the proportion of punishment that reflects each of the competing hypotheses for punishment mechanisms with the implementation of time pressure. Because the punishment motivation categories overlap, the total proportion of the bars exceeds the true proportion of punishment in the experiment. P-values are from random intercepts mixed effects models testing differences between the TP- (left, bars without crosses) and TP + (right, bars with white crosses) settings. Bars represent 95% confidence intervals of proportions.

We then evaluated if time pressure reduced the frequencies of any of the three justifiable mechanisms of punishment that we highlighted in Experiment 1. Results show that the frequency of the punishment decisions due to copying or retaliation did not change between the TP- and TP + settings (1.5% and 1.7% respectively, p = 0.893), the frequency of punishment for negative reinforcement did not change (3.5% and 3.5%, p = 0.634), the frequency of punishment for inequality aversion did not change (3.7% and 3.6%, p = 0.440), and the frequency of unclassified punishment did not change (1.3% and 1.1%, p = 0.645) (Fig. 4).

### Discussion

Although we successfully predicted that punishment would be slower than cooperation or defection, we did not observe the hypothesized reduction in punishment associated with time pressure, as game sessions from the TP + and TP- settings in Experiment 2 had a similar frequency of punishment decisions (6.3% vs. 6.5%, respectively). Furthermore, we found no substantial difference in frequency for any of the mechanisms for punishment when comparing game sessions with and without time pressure. We were particularly interested in observing a potential decrease in the frequency of punishment for negative reinforcement; previous studies have linked deliberation to the degradation of cooperation with others^54–56^, suggesting that limiting deliberation could potentially dissuade players from choosing the punishment option in favor of cooperation. However, we did not observe this decrease, a result which falls in line with other studies^30,38,57^ that limit their endorsement of the link between intuition and cooperation (and in our view, the link between deliberation and non-cooperation, which includes punishment).

In summary, time pressure did not substantially reduce the frequency of punishment decisions in general, potentially because prohibiting slow decisions does not address the specific mechanisms that drive punishment decisions. Since our investigation of external time pressure only addresses Hypothesis 1, further research to address Hypothesis 2 should be conducted by addressing each of the individual mechanisms for punishment in an experimental manner and evaluating how the distribution of decision-making changes.

## General discussion

Using two series of online, network-based public goods games, we found that on average, players spend more time when choosing to punish compared to choosing to cooperate or defect. Follow-up experimentation based on this result evaluated if experimental time pressure could reduce the occurrence of punishment in favor of cooperation; no such reduction occurred.

Our experimental efforts to determine if time pressure could reduce the occurrence of punishment were partially inspired by recent violent and damaging trends in the United States and around the world that reflect a widespread inclination to punish. We aimed to identify a practical method to suppress punishment or behavior that harms others in a social context; however, we were unable to do so. Reducing the occurrence of punishment is a difficult task which requires addressing the source of the initial motivation to punish.

We also investigated several potential mechanisms that drive punishment and observed a tendency for decision times to increase comparing punishment related to retaliation and punishment intended to promote future cooperation or reduce inequality. It is possible that players making decisions that are not retaliative in nature are considering and analyzing complex situations which would increase the time needed to process each part of the decision and therefore increase decision time. However, our experiments did not collect information from the players about the perceived complexity of their decisions. Future studies could take advantage of conducting interviews between rounds to investigate players’ perceptions and calculations regarding the decisions they made and the associated decision times.

Our study has several limitations that could limit the translation of our results to other settings in social and behavioral sciences. First, we did not measure several cultural, emotional, or psychological characteristics that could influence the relationship between punishment decision-making and decision speed in our experiments. Recent efforts analyzing the relationship between decision times and cooperation have explored how factors such as belief about other individuals’ intentions^58^, personal time preferences^59^, and age^60^ influence cooperation decision-making. Furthermore, recent work has found that the reputation of punishers decreases when they punish quickly, but when the punishment was slow, the punishers’ reputation instead increased^61^. Future studies should aim to evaluate these each of these characteristics in detail as they relate to punishment and decision times.

Second, we kept the payoff structure consistent across experimental settings with and without time pressure to evaluate the independent effect of time pressure. Our experimental payoff structure did not penalize punishers any more than cooperators were incentivized; however, this may not reflect realistic interactions in human society because choosing punishment is generally perceived as negative and may incur greater than expected costs for the punisher. It is possible that modifying the payoff structure to be more extreme (i.e., paying a greater cost to inflict the same level of harm shown in Experiments 1 and 2) could return greater differences in experimental participants’ decision-making profile.

Third, punishment in our experiments was indiscriminate and therefore unconditional (as focal players who chose punishment dealt the point-loss punishment to each connected player). However, in the real world, punishment is often conditional. Examples of conditional punishment include courts choosing to waive or lighten the punishment assigned to criminals if the criminals are first-time offenders or if the crimes occurred under extenuating circumstances or armies opting not to engage in battle if the costs to the combatants would exceed the benefits of victory. Future experiments based on our study’s framework could allow for conditional punishment options such as allowing players to choose between punishing all connected players and choosing to punish only individuals who were not cooperators in the prior round.

Finally, we recognize there are limitations associated with the use of MTurk workers to perform decision-making experiments. Several studies^28,29^ have noted shifts in the MTurk worker population which suggests that workers have learned ways to improve their financial output when engaging with decision-making games on the platform. Furthermore, the increasing cost of recruiting workers, especially to longer experimental sessions, limits the effective number of participants that can be recruited, leading to potential concerns about type II error. While we found that there was no statistical evidence for the ability of our time pressure condition to reduce the occurrence of punishment (or cooperation or defection), it is possible that the true effect was too small to detect given the number of participants we could recruit. Given these limitations, development of equivalence testing^62^ methods such as the two one-sided test (TOST) procedure that can be applied to clustered or multilevel data would be beneficial.

## Supplementary Information

Supplementary Information 1.

Supplementary Information 2.

Supplementary Information 3.

Supplementary Information 4.

## Data Availability

Data and statistical analysis code that support the results of this study are found in the Supplementary Materials.
