# Supplementary material for: Punishment is slower than cooperation or defection in online network games
Source: Sci Rep. 2024 Oct 3;14:23024. doi: 10.1038/s41598-024-72939-2 (PMC11449937; doi:10.1038/s41598-024-72939-2)
Supplement: Supplementary file 1 — Supplementary Information 1. [file 41598_2024_72939_MOESM1_ESM.docx]

**Supplementary Information**

| **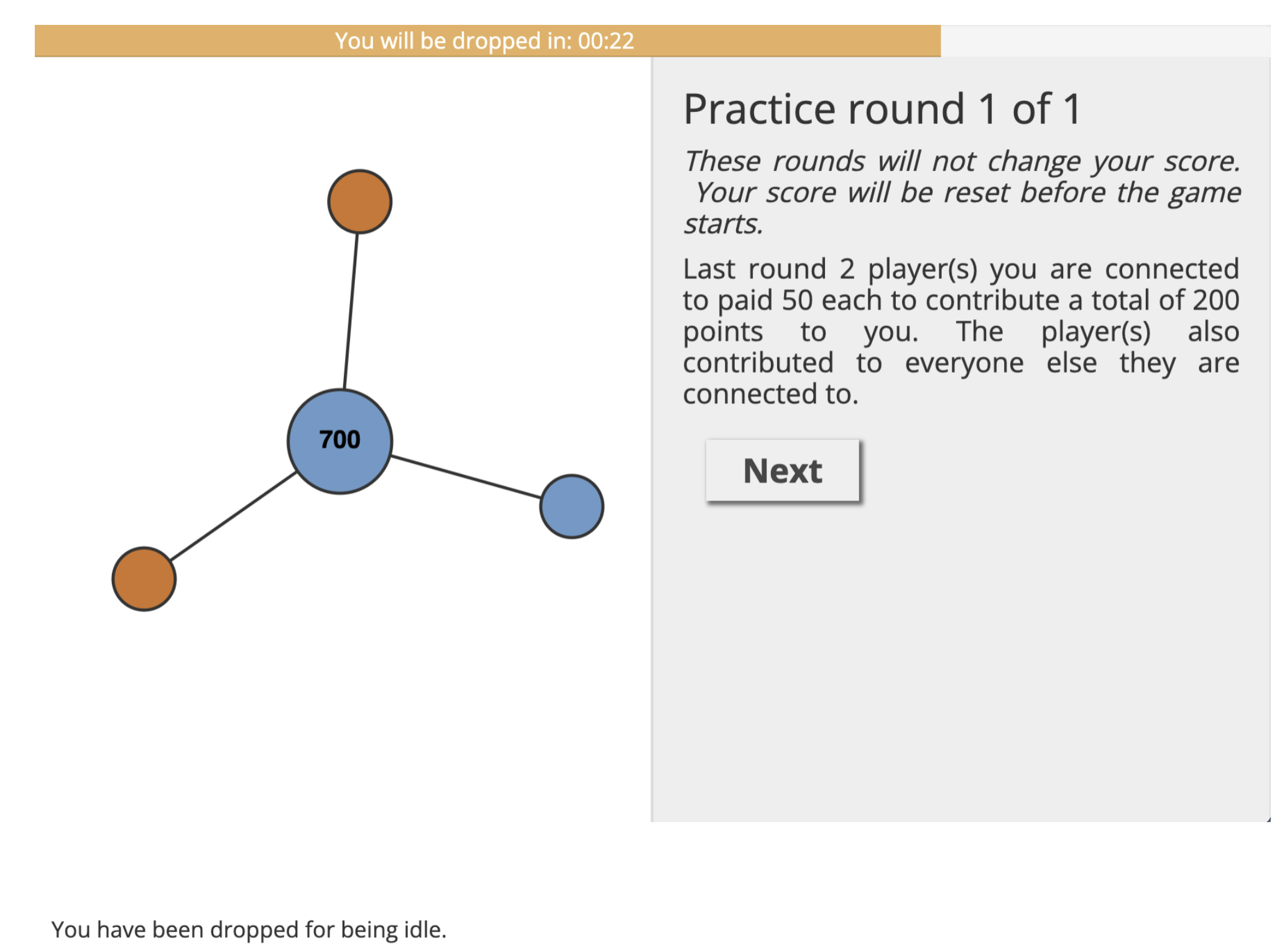** |
| --- |
| **Supplementary Figure S1**. **Example message shown to dropped players in the time pressure condition.** Players in the TP+ condition who did not click on a button within the allotted time were first given a warning (and their choice from the previous round was repeated). If players did not click in two different rounds, they were dropped from future rounds of the experiment and were shown the message “You were dropped for being idle.” |
| 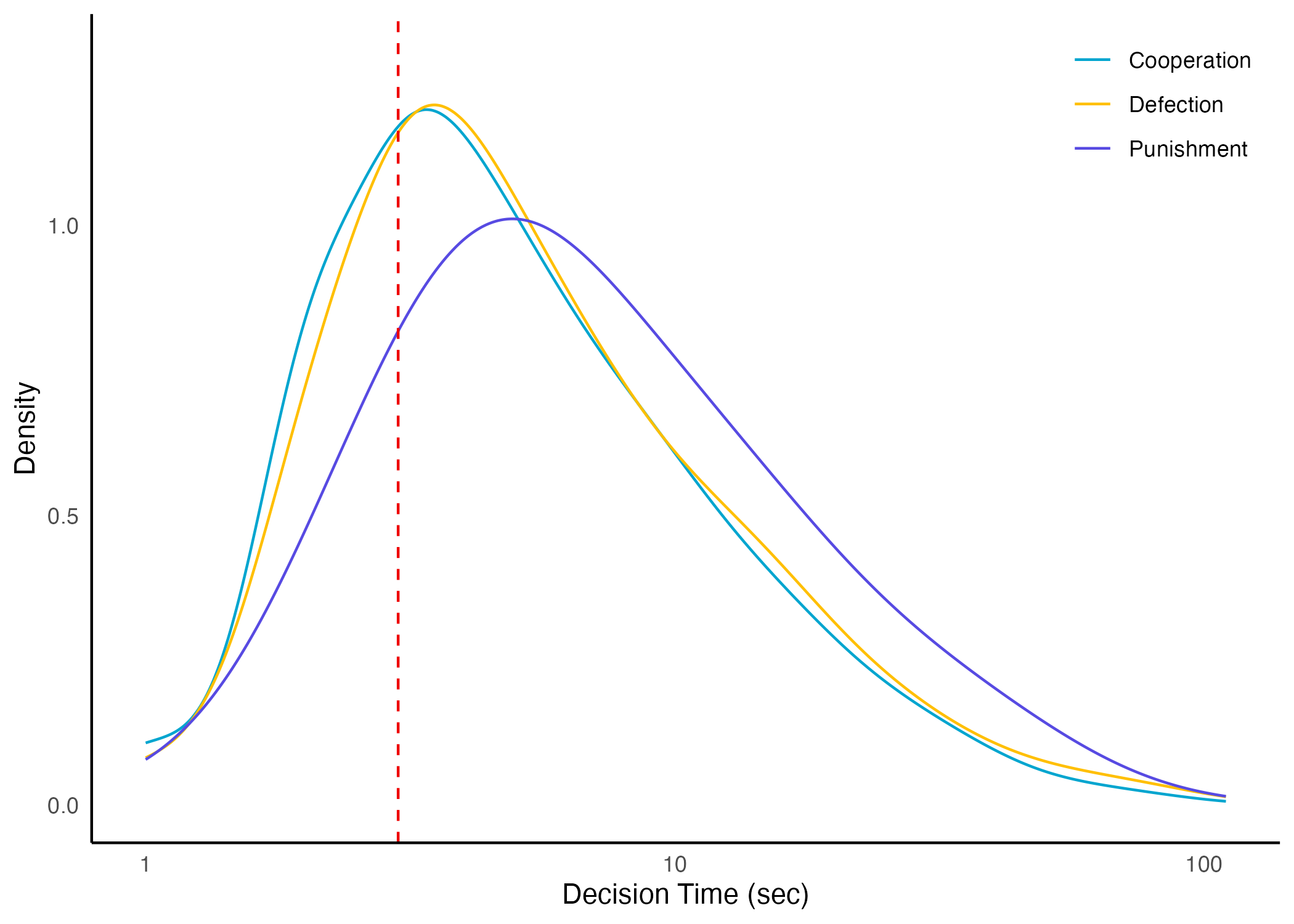 |
| **Supplementary Figure S2. Distributions of decision times for cooperation, defection, and punishment in Experiment 1**. The median decision times for cooperation and defection in Experiment 1 are close to three seconds (the red dashed line) while the median decision time for punishment is noticeably to the right of the red line. |

| 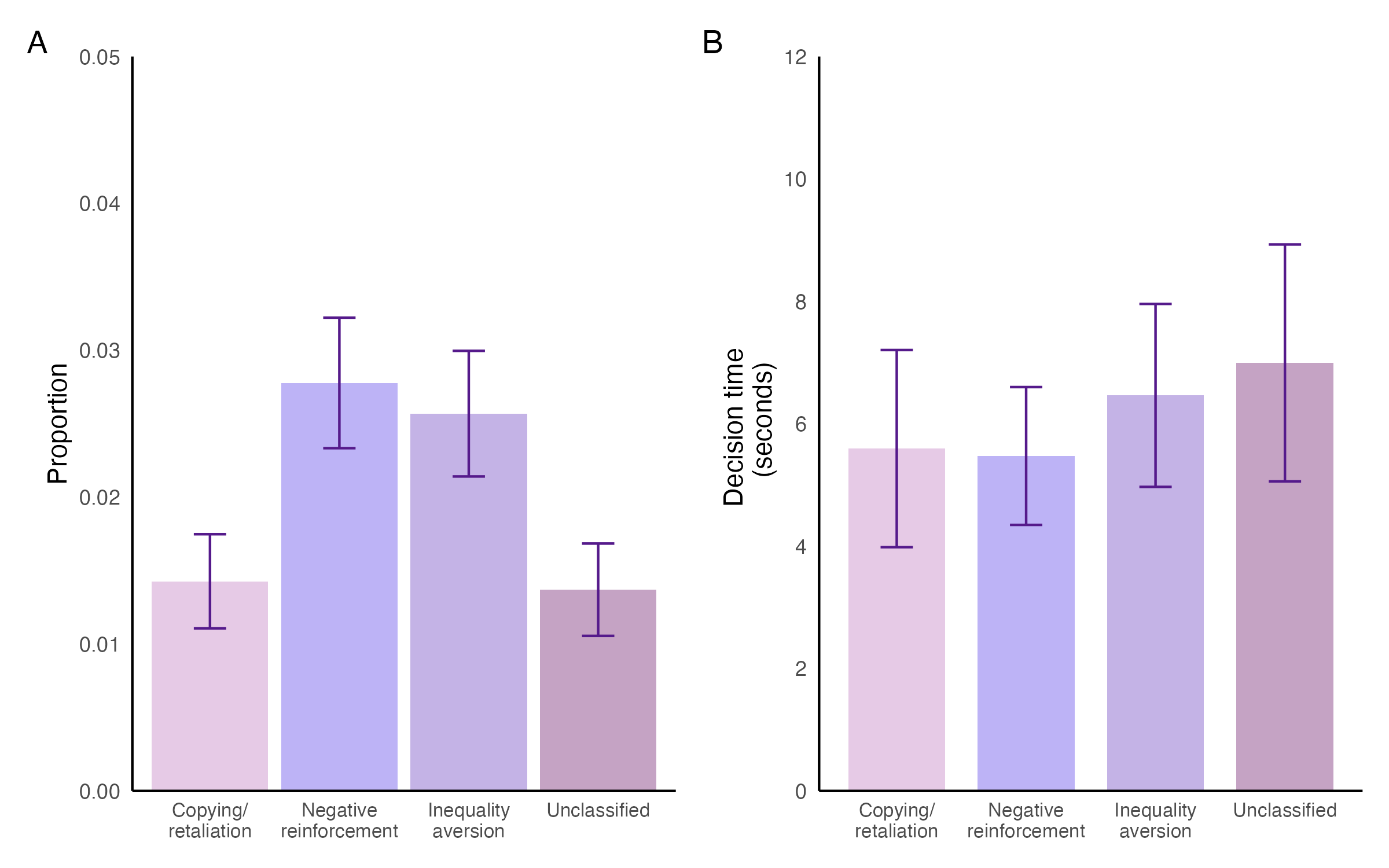 |
| --- |
| **Supplementary Figure S3. Distribution and decision times of punishment mechanisms in invisible-wealth games in Experiment 1. A**: The distribution of punishment mechanism types among invisible-wealth games in Experiment 1 mirrors the distribution of punishment mechanisms in the full Experiment 1 data. **B**. The general trend of increased decision times associated with inequality aversion or unclassified punishment compared to copying/retaliation or negative reinforcement punishment observed in the full Experiment 1 data holds when restricting data to only invisible-wealth games. Widening of the confidence intervals are expected after halving the sample size. Bars indicate 95% confidence intervals of proportions or decision time. |

|  | **Exp. 1** | **Exp. 2, TP-** | **Exp. 2, TP+** |
| --- | --- | --- | --- |
| N | 9,776 | 5,247 | 4,066 |
| **Fixed Effects** |  |  |  |
| Defection vs. cooperation | 0.30411 (0.23036) [p = 0.18684] | -0.273991 (0.106943) [p = 0.01048] | -0.051397 (0.01966) [p = 0.0089] |
| Punishment vs. cooperation | 1.21569 (0.42149) [p = 0.00393] | 0.47002 (0.147547) [p = 0.00145] | 0.115546 (0.02799) [p = 0.000037] |
| Round | -0.16708 (0.01916) [p < 0.0001] | -0.020730 (0.006745) [p = 0.00213] | -0.006124 (0.00109) [p < 0.0001] |
| Intercept | 6.79184 (0.30462) [p < 0.0001] | 3.329949 (0.122798) [p < 0.0001] | 2.090189 (0.023485) [p < 0.0001] |
| **Random Effects** |  |  |  |
| Player-level variance | 11.789 (3.434) | 1.58559 (1.2592) | 0.07370 (0.27147) |
| Game-level variance | 1.824 (1.350) | 0.08172 (0.2859) | 0.00315 (0.05613) |
| Residual variance | 64.809 (8.050) | 4.38568 (2.0942) | 0.08420 (0.29018) |

**Supplementary Table S1. Multilevel random intercepts models for decision times for all 3 experimental settings.**  The reference category for decision type was cooperation. Standard errors for fixed effects and standard deviations for random effects are shown in parentheses. P-values are shown in square brackets.

|  | **Punishment** | **Cooperation** | **Defection** |
| --- | --- | --- | --- |
| N | 10,654 | 10,654 | 10,654 |
| **Fixed Effects** |  |  |  |
| Time Pressure | 0.77967 (0.39389, 1.54330) [p = 0.475] | 1.30413 (0.39811, 4.27207) [p = 0.661] | 0.80540 (0.20294, 3.19637) [p = 0.758] |
| Round | 0.97390 (0.95276, 0.99551) [p = 0.182] | 0.9530 (0.93636, 0.96993) [p < 0.00001] | 1.05696 (1.03725, 1.07704) [p < 0.00001] |
| Intercept | 0.02495 (0.01506, 0.04132) [p < 0.00001] | 0.44260 (0.18905, 1.03621) [p = 0.0604] | 1.03491 (0.38632, 2.77243) [p = 0.94558] |
| **Random Effects** |  |  |  |
| Player-level variance | 6.908 (2.6283) | 27.00 (5.196) | 35.06 (5.921) |
| Game-level variance | 0.641 (0.8006) | 2.42 (1.556) | 3.392 (1.842) |

**Supplementary Table S2: Multilevel logistic random intercepts model for the effect of time pressure on the odds of punishment, cooperation, and defection in Experiment 2 (results for Fig. 3A).** Estimates shown are exponentiated and represent odds ratios. 95% confidence intervals for fixed effects odds ratios and standard deviations of random effects are shown in parentheses. P-values are shown in square brackets.

|  | **Copying/**  **retaliation** | **Negative reinforcement** | **Inequality**  **aversion** | **Unclassified** |
| --- | --- | --- | --- | --- |
| N | 10,654 | 10,654 | 10,654 | 10,654 |
| **Fixed Effects** |  |  |  |  |
| Time Pressure | 0.93220 (0.34538, 2.51606) [p = 0.889] | 0.83141 (0.39301, 1.75886) [p = 0.629] | 0.76709 (0.39435, 1.49212) [p = 0.435] | 0.85826 (0.44474, 1.6524) [p = 0.649] |
| Round | 1.02502 (0.98436, 1.0580) [p = 0.269] | 1.03644 (1.00987, 1.063471) [p = 0.0069] | 1.02228 (0.99594, 1.04932) [p = 0.098] | 0.83194 (0.79264, 0.87319) [p < 0.00001] |
| Intercept | 0.00575 (0.00269, 0.01228) [p < 0.00001] | 0.01049 (0.00594, 0.01853) [p < 0.00001] | 0.01042 (0.00626, 0.01733) [p < 0.00001] | 0.02098 (0.01249, 0.03523) [p < 0.00001] |
| **Random Effects** |  |  |  |  |
| Player-level variance | 2.722 (1.650) | 4.903 (2.214) | 6.9448 (2.6374) | 3.919 (1.9798) |
| Game-level variance | 2.117 (1.455) | 1.007 (1.004) | 0.4017 (0.6338) | 0.358 (0.5984) |

**Supplementary Table S3: Multilevel logistic random intercepts model for the effect of time pressure on the odds of individual punishment mechanisms in Experiment 2 (results for Fig. 4).** Estimates shown are exponentiated and represent odds ratios. 95% confidence intervals for fixed effects odds ratios and standard deviations of random effects are shown in parentheses. P-values are shown in square brackets.

|  | **Exp. 1** |
| --- | --- |
| N | 508 |
| **Fixed Effects** |  |
| Negative reinforcement | 0.5307 (1.1866) [p = 0.655] |
| Inequality aversion | 1.5698 (1.1200) [p = 0.162] |
| Unclassified | 3.7815 (1.6083) [p = 0.019] |
| Round | -0.1209 (0.1023) [p = 0.2377] |
| Intercept | 6.2481 (1.5349) [p < 0.0001] |
| **Random Effects** |  |
| Player-level variance | 23.47 (4.845) |
| Game-level variance | 11.29 (3.360) |
| Residual variance | 64.44 (8.028) |

**Supplementary Table S4. Multilevel random intercepts models for decision times to compare individual punishment mechanisms.**  The reference category for decision type was punishment for copying/retaliation. Standard errors for fixed effects and standard deviations for random effects are shown in parentheses. P-values are shown in square brackets.

|  | **Exp. 1** |
| --- | --- |
| N | 508 |
| **Fixed Effects** |  |
| Last punished | -2.4355 (0.9306) [p = 0.0091] |
| Round | -0.1489 (0.1003) [p = 0.1384] |
| Intercept | 9.1691 (1.1074) [p < 0.00001] |
| **Random Effects** |  |
| Player-level variance | 23.89 (4.887) |
| Game-level variance | 10.43 (3.229) |
| Residual variance | 64.10 (8.006) |

**Supplementary Table S5: Multilevel random intercepts models for the effect of having been punished in the prior round on punishment decision times in Experiment 1.** Standard errors for fixed effects and standard deviations for random effects are shown in parentheses. P-values are shown in square brackets.

|  | **Exp. 1** |
| --- | --- |
| N | 9020 |
| **Fixed Effects** |  |
| Punishment | 1.2756 (0.4545) [p = 0.0050] |
| Prior round rate of punishment | -0.6620 (0.8331) [p = 0.4268] |
| Punishment x prior round rate of punishment | -4.0876 (2.4373) [p = 0.0936] |
| Round | -0.1310 (0.0206) [p < 0.0001] |
| Intercept | 6.5745 (0.2951) [p < 0.0001]] |
| **Random Effects** |  |
| Player-level variance | 11.076 (3.328) |
| Game-level variance | 1.554 (1.247) |
| Residual variance | 59.983 (7.745) |

**Supplementary Table S6. Multilevel random intercepts model for the effect of the interaction between prior round rate of punishment and choosing to punish on decision times in Experiment 1.** Standard errors for fixed effects and standard deviations for random effects are shown in parentheses. P-values are shown in square brackets.

|  | **Exp. 1** | **Exp. 2, TP-** | **Exp. 2, TP+** |
| --- | --- | --- | --- |
| N | 9,776 | 5,247 | 4,066 |
| **Fixed Effects** |  |  |  |
| Defection vs. cooperation | 0.30663 (0.23045) [p = 0.18339] | -0.274949 (0.106999) [p = 0.01025] | -0.051276 (0.01966) [p = 0.00918] |
| Punishment vs. cooperation | 1.21876 (0.42156) [p = 0.00385] | 0.468723 (0.147570) [p = 0.00150] | 0.115836 (0.02799) [p = 0.000036] |
| Low point allocation | 0.17879 (0.34334) [p = 0.60275] | 0.085236 (0.158290) [p = 0.59060] | 0.020266 (0.032963) [p = 0.53909] |
| Round | -0.16710 (0.01916) [p < 0.0001] | -0.020717 (0.006745) [p = 0.00214] | -0.006126 (0.00109) [p < 0.0001] |
| Intercept | 6.66573 (0.38930) [p < 0.0001] | 3.272191 (0.164006) [p < 0.0001] | 2.076096 (0.032851) [p < 0.0001] |
| **Random Effects** |  |  |  |
| Player-level variance | 11.806 (3.436) | 1.58849 (1.2604) | 0.07384 (0.27174) |
| Game-level variance | 1.826 (1.351) | 0.08541 (0.2922) | 0.00320 (0.05653) |
| Residual variance | 64.809 (8.050) | 4.38547 (2.0942) | 0.08420 (0.29017) |

**Supplementary Table S7. Multilevel random intercepts models for decision times adjusting for point allocation for all 3 experimental settings.**  The reference category for decision type was cooperation. Point allocation was a binary condition (either high or low). Standard errors for fixed effects and standard deviations for random effects are shown in parentheses. P-values are shown in square brackets.

|  | **Exp. 1** | **Exp. 2, TP-** | **Exp. 2, TP+** |
| --- | --- | --- | --- |
| N | 508 | 341 | 184 |
| **Fixed Effects** |  |  |  |
| Negative reinforcement | 0.5899 (1.1890) [p = 0.620] | 1.0346 (0.8808) [p = 0.0411] | 0.02436 (0.0684) [p = 0.722] |
| Inequality aversion | 1.2323 (1.2123) [p = 0.3100] | -0.1425 (0.5918) [p = 0.8099] | -0.0252 (0.0741) [p = 0.734] |
| Unclassified | 3.7892 (1.6085) [p = 0.0190] | 0.3789 (0.7157) [p = 0.5970] | -0.0011 (0.0976) [p = 0.991] |
| Low point allocation | 1.0097 (1.3814) [p = 0.4663] | -0.5789 (0.7353) [p = 0.4342] | 0.0191 (0.1028) [p = 0.853] |
| Round | -0.1250 (0.1024) [p = 0.2226] | -0.0355 (0.0448) [p = 0.4287] | -0.00424 (0.00603) [p = 0.483] |
| Intercept | 5.7683 (1.6748) [p < 0.0001] | 4.1057 (0.8808) [p < 0.0001] | 2.185 (0.1166) [p < 0.0001] |
| **Random Effects** |  |  |  |
| Player-level variance | 24.29 (4.928) | 6.570 (2.563) | 0.1106 (0.3326) |
| Game-level variance | 11.12 (3.335) | 0 (0) | 0.00683 (0.08265) |
| Residual variance | 64.20 (8.013) | 9.037 (3.006) | 0.0764 (0.2764) |

**Supplementary Table S8. Multilevel random intercepts models for decision times to compare individual punishment mechanisms adjusting for point allocation.**  The reference category for decision type was punishment for copying/retaliation. Point allocation was a binary condition (either high or low). Standard errors for fixed effects and standard deviations for random effects are shown in parentheses. P-values are shown in square brackets.

|  | **Exp. 1** | **Exp. 2, TP-** | **Exp. 2, TP+** |
| --- | --- | --- | --- |
| N | 283 | 211 | 113 |
| **Fixed Effects** |  |  |  |
| Local rate of defection in prior round > 0.5 | 1.7695 (1.2852) [p = 0.170] | 1.2505 (0.5954) [p = 0.037] | 0.0953 (0.0662) [p = 0.153] |
| Round | -0.0743 (0.1413) [p = 0.599] | -0.03402 (0.0711) [p = 0.603] | -0.0021 (0.0086) [p = 0.804] |
| Intercept | 7.0056 (1.5262) [p < 0.0001] | 3.5290 (0.7470) [p < 0.0001] | 2.117 (0.0980) [p < 0.0001] |
| **Random Effects** |  |  |  |
| Player-level variance | 0 (0) | 6.415 (2.533) | 0.1242 (0.3525) |
| Game-level variance | 29.43 (5.425) | 0.0094 (0.0970) | 0 (0) |
| Residual variance | 66.96 (8.183) | 12.46 (3.529) | 0.0691 (0.263) |

**Supplementary Table S9. Multilevel random intercepts models for decision times for inequality aversion punishment decisions adjusting for past round defection rate.** The reference category for local rate of defection in the prior round was a local rate ≤ 0.5. Standard errors for fixed effects and standard deviations for random effects are shown in parentheses. P-values are shown in square brackets.

|  | **Exp. 1** |
| --- | --- |
| N | 513 |
| **Fixed Effects** |  |
| Visible wealth | -0.1753 (0.5677) [p = 0.757] |
| Round | 0.0418 (0.0374) [p = 0.264] |
| Intercept | 0.3369 (0.4962) [p = 0.497] |
| **Random Effects** |  |
| Player-level variance | 9.220 (3.036) |
| Game-level variance | 0 (0) |

**Supplementary Table S10. Multilevel logistic random intercepts model for the effect of wealth visibility on the odds of inequality aversion punishment among punishment decisions.** The reference category for the visible wealth variable is no wealth visibility. Standard errors for fixed effects and standard deviations for random effects are shown in parentheses. P-values are shown in square brackets.

|  | **Exp. 1** |
| --- | --- |
| N | 286 |
| **Fixed Effects** |  |
| Wealth visibility | 1.0947 (1.9938) [p = 0.5864] |
| Round | -0.01564 (0.1389) p = 0.9104] |
| Intercept | 7.1625 (1.7826) [p = 0.00015] |
| **Random Effects** |  |
| Player-level variance | 0 (0) |
| Game-level variance | 25.92 (5.092) |
| Residual variance | 71.95 (8.482) |

**Supplementary Table S11. Multilevel random intercepts model for the effect of wealth visibility on the decision times of inequality aversion punishment decisions.** The reference category for the visible wealth variable is no wealth visibility. Standard errors for fixed effects and standard deviations for random effects are shown in parentheses. P-values are shown in square brackets.

|  | **Exp. 1** | **Exp. 2, TP-** | **Exp. 2, TP+** |
| --- | --- | --- | --- |
| N | 4,017 | 2,693 | 2,174 |
| **Fixed Effects** |  |  |  |
| Had a prior round defector | -0.4443 (0.5473) [p = 0.4084] | -0.0059 (0.1662) [p = 0.972] | -0.0006 (0.0349) [p = 0.987] |
| Round | -0.1214 (0.0346) [p = 0.0004] | -0.0146 (0.0084) [p = 0.082] | -0.0070 (0.0015) [p < 0.0001] |
| Intercept | 7.371 (0.6197) [p < 0.00001] | 3.0819 (0.1933) [p < 0.00001] | 2.0395 (0.0462) [p < 0.000001] |
| **Random Effects** |  |  |  |
| Player-level variance | 15.075 (3.883) | 1.460 (1.208) | 0.075 (0.274) |
| Game-level variance | 0.865 (0.930) | 0 (0) | 0.011 (0.106) |
| Residual variance | 70.638 (8.405) | 2.951 (1.718) | 0.079 (0.281) |

**Supplementary Table S12. Multilevel random intercepts models for decision times for retaliative defections.** Defections were considered retaliative if the player had a defector in their local environment in the past round. The reference category for local rate of defection in the prior round was a local rate ≤ 0.5. Standard errors for fixed effects and standard deviations for random effects are shown in parentheses. P-values are shown in square brackets.

|  | **Exp. 1** | **Exp. 2, TP-** | **Exp. 2, TP+** |
| --- | --- | --- | --- |
| N | 9,776 | 5,247 | 5,407 |
| **Fixed Effects** |  |  |  |
| Punishment | 0.9536 (0.1141) [p = 0.691] | 1.3454 (0.2656) [p = 0.1329] | 1.1248 (0.2481) [p = 0.5940] |
| Round | 0.9002 (0.0052) [p < 0.00001] | 0.9448 (0.0086) [p < 0.00001] | 0.9408 (0.0089) [p < 0.00001] |
| Intercept | 5.2865 (0.5781) [p < 0.00001] | 8.1130 (1.0850) [p < 0.00001] | 9.2522 (1.7397) [p < 0.00001] |
| **Random Effects** |  |  |  |
| Player-level variance | 0.6461 (0.8038) | 2.1305 (1.460) | 2.692 (1.641) |
| Game-level variance | 0.4029 (0.6347) | 0.0998 (0.3159) | 0.4752 (0.6893) |

**Supplementary Table S13. Multilevel logistic random intercepts models for the effect of choosing punishment on rewiring.** Estimates shown are exponentiated and represent odds ratios. 95% confidence intervals for fixed effects odds ratios and standard deviations of random effects are shown in parentheses. P-values are shown in square brackets.
