# Supplementary material for: Punishment is slower than cooperation or defection in online network games
Source: Sci Rep. 2024 Oct 3;14:23024. doi: 10.1038/s41598-024-72939-2 (PMC11449937; doi:10.1038/s41598-024-72939-2)
Supplement: Supplementary file 2 — Supplementary Information 2. [file 41598_2024_72939_MOESM2_ESM.pdf]

Data Analysis for Punishment is Slower than Cooperation or Defection

Table of contents

1. Setup **2**
Load required packages 2
Load required data 2
Create helper functions 3

2. Analysis **3**
Number of observations per experiment 3
Per-game characteristics 3
Distribution of decision-making 5
Network characteristics 7
Decision times 8

3. Regression Modeling **9**
Punishment vs. time pressure 9
Cooperation vs. time pressure 10
Defection vs. time pressure 12
Decision times vs. time pressure (not useful) 13
Punishment mechanisms vs. time pressure 16
Decision time as the outcome 20
Supplementary Analyses for Verification 29
Testing the reciprocal effect of defection for defection decisions 42
Evaluating the effect of choosing punishment on rewiring 45

4. Figures **46**
Figure 1 - Example of Player's Screen 46
Figure 2 - Behavior Distribution, Decision Times, and Punishment Mechanisms, Experiment 1 47
Main 54

Figure 3 - Behavior Distribution, Decision Times, and Punishment Mechanisms, Experiment 2 . . . 55
Main . . . 56
Figure 4 - Punishment Mechanism Decision Times, Experiment 2 . . . 59
Main . . . 64
Figure S2 - Distribution of Decision Times, Experiment 1 . . . 66
Figure S3 - Sensitivity Analysis for Invisible Wealth Games Only (Exp. 1) . . . 67

1. Setup

Load required packages

```
library(tidyverse)
library(igraph)
library(lme4)
library(lmerTest)
library(DescTools)
library(data.table)
library(patchwork)
library(stringr)
library(ggpattern)
library(magick)
library(broom.mixed)
library(simr)
```

Load required data

```
## Experiment 1
exp1data = read_csv("~/Documents/Projects/harming_esn/data/final/exp1data_rev3.csv",
                    show_col_types = FALSE)

## Experiment 2
exp2data = read_csv("~/Documents/Projects/harming_esn/data/final/exp2data_rev3.csv",
                    show_col_types = FALSE)
```

Create helper functions

```
mean1 = function(x) {mean(x,na.rm=TRUE)}
median1 = function(x) {median(x, na.rm = TRUE)}
sd1 = function(x) {sd(x, na.rm = TRUE)}
se_mean = function(x) sd1(x)/sqrt(sum(is.na(x) == 0))
```

2. Analysis

Number of observations per experiment

Experiment 1

```
# Experiment 1
exp1data %>%
  filter(round > 0, is.na(behavior) == F) %>%
  nrow()
```

```
[1] 9776
```

Experiment 2

```
# Experiment 2
exp2data %>%
  filter(round > 0, is.na(behavior) == F) %>%
  nrow()
```

```
[1] 10654
```

Per-game characteristics

Experiment 1

```
exp1data %>%
  filter(round >= 1) %>%
  group_by(game) %>%
  select(superid) %>%
  unique() %>%
  nrow()
```

```
[1] 719
```

```
exp1data %>%
  filter(round >= 1) %>%
  group_by(game) %>%
  select(superid) %>%
  unique() %>%
  summarize(n = n()) %>%
  summarize(`Mean Players` = mean(n),
            `Min Players` = min(n),
            `Max Players` = max(n))
```

```
# A tibble: 1 x 3
  `Mean Players` `Min Players` `Max Players`
           <dbl>         <int>         <int>
1           14.4             8            25
```

Experiment 2

```
# Players per game, min, max - Experiment 2
exp2data %>%
  filter(round >=0) %>%
  group_by(game) %>%
  select(superid) %>%
  unique() %>%
  summarize(n = n()) %>%
  summarize(`Mean Players` = mean(n),
            `Min Players` = min(n),
            `Max Players` = max(n))
```

```
# A tibble: 1 x 3
  `Mean Players` `Min Players` `Max Players`
           <dbl>         <int>         <int>
1           14.8             8            20
```

Number of observations in Exp. 2, TP+/TP- settings

```
exp2data %>%
  filter(time_pressure == "Plus", round >= 1, behavior %in% c("C", "D", "P")) %>%
  nrow()
```

```
[1] 5407
```

```
exp2data %>%
  filter(time_pressure == "Minus", round >= 1, behavior %in% c("C", "D", "P")) %>%
  nrow()
```

```
[1] 5247
```

```
# Number of players per condition in Experiment 2
exp2data %>%
  filter(round >= 1, behavior %in% c("C", "D", "P")) %>%
  group_by(time_pressure) %>%
  select(superid) %>%
  unique() %>%
  count()
```

```
Adding missing grouping variables: `time_pressure`
```

```
# A tibble: 2 x 2
# Groups:   time_pressure [2]
  time_pressure     n
  <chr>         <int>
1 Minus           366
2 Plus            372
```

Distribution of decision-making

Experiment 1

```
# Decision distribution - Experiment 1
data1_behavior_count = exp1data %>%
  filter(round >= 1) %>%
  group_by(behavior) %>%
  filter(behavior %in% c("C", "D", "P")) %>%
  summarize(count = n()) %>%
  ungroup() %>%
  mutate(proportion = count/sum(count))

# Confidence Intervals
data1_behavior_CI = MultinomCI(x = c(4878, 4336, 562), sides = "two.sided") %>%
  as_tibble()
```

Experiment 2

```
# Decision distribution - Experiment 2
exp2data_count = exp2data %>% filter(round >= 1) %>%
  group_by(behavior) %>%
  filter(behavior %in% c("C", "D", "P")) %>%
  summarize(count = n()) %>%
  ungroup() %>%
  mutate(proportion = count/sum(count))

exp2data_all_CI = MultinomCI(c(4185, 5790, 679), sides = "two.sided")
```

```
exp2data_tp_plus_count = exp2data %>%
  group_by(behavior) %>%
  filter(time_pressure == "Plus", behavior %in% c("C", "D", "P")) %>%
  summarize(count = n()) %>%
  ungroup() %>%
  mutate(proportion = count/sum(count))

exp2data_tp_plus_CI = MultinomCI(c(2172, 2897, 338), sides = "two.sided")

exp2data_tp_plus_times = exp2data %>%
  group_by(behavior) %>%
  filter(time_pressure == "Plus", behavior %in% c("C", "D", "P")) %>%
  summarize(mean_dt = mean1(behaviorTime_sec),
            se_mean_dt = se_mean(behaviorTime_sec),
            LL_mean = mean_dt - 1.96*se_mean_dt,
            UL_mean = mean_dt + 1.96*se_mean_dt)

exp2data_tp_minus_count = exp2data %>%
  group_by(behavior) %>%
  filter(time_pressure == "Minus", behavior %in% c("C", "D", "P")) %>%
  summarize(count = n()) %>%
  ungroup() %>%
  mutate(proportion = count/sum(count))

exp2data_tp_minus_times = exp2data %>%
  group_by(behavior) %>%
  filter(time_pressure == "Minus", behavior %in% c("C", "D", "P")) %>%
  summarize(mean_dt = mean1(behaviorTime_sec),
            se_mean_dt = se_mean(behaviorTime_sec),
            LL_mean = mean_dt - 1.96*se_mean_dt,
            UL_mean = mean_dt + 1.96*se_mean_dt)
```

```
exp2data_tp_minus_CI = MultinomCI(c(2013, 2893, 341), sides = "two.sided")
```

```
# Behavior breakdown - Experiment B, TP-
exp2data_tp_minus_count = exp2data %>%
  group_by(behavior) %>%
  filter(time_pressure == "Minus", behavior %in% c("C", "D", "P")) %>%
  summarize(count = n()) %>%
  ungroup() %>%
  mutate(proportion = count/sum(count))

exp2data_tp_minus_CI = MultinomCI(c(2013, 2893, 341), sides = "two.sided")
```

```
# Behavior breakdown - Experiment B, TP+
exp2data_tp_plus_count = exp2data %>%
  group_by(behavior) %>%
  filter(time_pressure == "Plus", behavior %in% c("C", "D", "P")) %>%
  summarize(count = n()) %>%
  ungroup() %>%
  mutate(proportion = count/sum(count))

exp2data_tp_plus_CI = MultinomCI(c(2172, 2897, 338), sides = "two.sided")
```

Network characteristics

Experiment 1

```
mean1(exp1data$degree)
```

```
[1] 5.911319
```

```
min(exp1data$degree, na.rm = T)
```

```
[1] 1
```

```
max(exp1data$degree, na.rm = T)
```

```
[1] 17
```

Experiment 2

```
mean1(exp2data$degree)
```

```
[1] 5.721265
```

```
min(exp2data$degree, na.rm = T)
```

```
[1] 1
```

```
max(exp2data$degree, na.rm = T)
```

```
[1] 16
```

Decision times

Experiment 1

```
data1_times = exp1data %>%
  group_by(behavior) %>%
  filter(behavior %in% c("C", "D", "P")) %>%
  summarize(mean_dt = mean1(behaviorTime_sec),
            se_mean_dt = se_mean(behaviorTime_sec),
            LL_mean = mean_dt - 1.96*se_mean_dt,
            UL_mean = mean_dt + 1.96*se_mean_dt)
```

```
exp1data %>%
  filter(local_rate_punish_lag > 0.25, behavior %in% c("C", "D", "P")) %>%
  group_by(behavior) %>%
  summarize(mean_dt = mean1(behaviorTime_sec),
            se_mean_dt = se_mean(behaviorTime_sec),
            LL_mean = mean_dt - 1.96*se_mean_dt,
            UL_mean = mean_dt + 1.96*se_mean_dt)
```

```
# A tibble: 3 x 5
  behavior mean_dt se_mean_dt LL_mean UL_mean
  <chr>      <dbl>      <dbl>   <dbl>   <dbl>
1 C           7.19      1.05     5.14    9.24
2 D           4.57      0.298    3.99    5.16
3 P           5.45      1.19     3.12    7.77
```

Experiment 2

```
exp2data %>%
  group_by(behavior, time_pressure) %>%
  filter(behavior %in% c("C", "D", "P")) %>%
  summarize(mean_dt = mean1(behaviorTime_sec),
            se_mean_dt = se_mean(behaviorTime_sec),
            UL_mean = mean_dt + 1.96*se_mean_dt,
            LL_mean = mean_dt - 1.96*se_mean_dt)
```

```
`summarise()` has grouped output by 'behavior'. You can override using the
`.groups` argument.
```

```
# A tibble: 6 x 6
# Groups:   behavior [3]
  behavior time_pressure mean_dt se_mean_dt UL_mean LL_mean
  <chr>    <chr>           <dbl>      <dbl>   <dbl>   <dbl>
1 C        Minus            3.13    0.0537     3.24    3.03
2 C        Plus             2.02    0.00987    2.04    2.00
3 D        Minus            2.85    0.0419     2.93    2.77
4 D        Plus             1.92    0.00826    1.94    1.91
5 P        Minus            3.82    0.203      4.22    3.42
6 P        Plus             2.11    0.0301     2.17    2.05
```

3. Regression Modeling

Punishment vs. time pressure

```
m1 = glmer(behavior_punish ~ time_pressure + round + (1|game) + (1|superid),
           data = exp2data %>% filter(round > 0), family = binomial, nAGQ=0,
           control = glmerControl(optimizer = c("bobyqa"),
                                  optCtrl=list(maxfun=2e5),
                                  calc.derivs=FALSE))
summary(m1)
```

```
Generalized linear mixed model fit by maximum likelihood (Adaptive
  Gauss-Hermite Quadrature, nAGQ = 0) [glmerMod]
 Family: binomial  ( logit )
Formula: behavior_punish ~ time_pressure + round + (1 | game) + (1 | superid)
```

```
   Data: exp2data %>% filter(round > 0)
Control: glmerControl(optimizer = c("bobyqa"), optCtrl = list(maxfun = 2e+05),
    calc.derivs = FALSE)

     AIC      BIC   logLik deviance df.resid
  3639.8   3676.2  -1814.9   3629.8    10742

Scaled residuals:
    Min      1Q  Median      3Q     Max
-2.9801 -0.1020 -0.0884 -0.0793  4.6005

Random effects:
 Groups  Name        Variance Std.Dev.
 superid (Intercept) 6.908    2.6283
 game    (Intercept) 0.641    0.8006
Number of obs: 10747, groups:  superid, 739; game, 50

Fixed effects:
                  Estimate Std. Error z value Pr(>|z|)
(Intercept)       -3.69099    0.25747 -14.335   <2e-16 ***
time_pressurePlus -0.24888    0.34838  -0.714   0.4750
round             -0.02644    0.01120  -2.362   0.0182 *
---
Signif. codes:  0 '***' 0.001 '**' 0.01 '*' 0.05 '.' 0.1 ' ' 1

Correlation of Fixed Effects:
            (Intr) tm_prP
tm_prssrPls -0.661
round       -0.324 -0.001
```

```
# p = 0.475
```

Cooperation vs. time pressure

```
m1.1 = glmer(behavior_coop ~ time_pressure + round + (1|game) + (1|superid),
             data = exp2data %>% filter(round > 0), family = binomial, nAGQ=0,
             control = glmerControl(optimizer = c("bobyqa"),
                                    optCtrl=list(maxfun=2e5),
                                    calc.derivs=FALSE))
summary(m1.1)
```

```
Generalized linear mixed model fit by maximum likelihood (Adaptive
  Gauss-Hermite Quadrature, nAGQ = 0) [glmerMod]
 Family: binomial  ( logit )
Formula: behavior_coop ~ time_pressure + round + (1 | game) + (1 | superid)
   Data: exp2data %>% filter(round > 0)
Control: glmerControl(optimizer = c("bobyqa"), optCtrl = list(maxfun = 2e+05),
    calc.derivs = FALSE)

     AIC      BIC   logLik deviance df.resid
  6102.1   6138.5  -3046.0   6092.1    10742

Scaled residuals:
    Min      1Q  Median      3Q     Max
-4.2875 -0.1092 -0.0839  0.1164  4.4511

Random effects:
 Groups  Name        Variance Std.Dev.
 superid (Intercept) 27.00    5.196
 game    (Intercept)  2.42    1.556
Number of obs: 10747, groups:  superid, 739; game, 50

Fixed effects:
                  Estimate Std. Error z value Pr(>|z|)
(Intercept)      -0.815089   0.434020  -1.878   0.0604 .
time_pressurePlus  0.265534   0.605401   0.439   0.6609
round            -0.048143   0.008987  -5.357 8.46e-08 ***
---
Signif. codes:  0 '***' 0.001 '**' 0.01 '*' 0.05 '.' 0.1 ' ' 1

Correlation of Fixed Effects:
            (Intr) tm_prP
tm_prssrPls -0.699
round       -0.157 -0.002
```

```
# p = 0.661
```

Power calculation for Experiment 2

We are interested here in the power of Experiment 2 to detect differences in the rate of cooperation. A simulation-based approach for determining power is used due to the hierarchical structure fo the experimental data.

```r
# Use model 1.1 as the baseline
m1.1_pwr = m1.1
round(summary(m1.1_pwr)$coef, 4)
```

```
                  Estimate Std. Error z value Pr(>|z|)
(Intercept)        -0.8151     0.4340 -1.8780   0.0604
time_pressurePlus   0.2655     0.6054  0.4386   0.6609
round              -0.0481     0.0090 -5.3571   0.0000
```

We want to estimate the ability of Exp. 2 to determine an 11.7% increase in cooperation relative to the deliberative condition. In this case, the deliberative condition is the TP- setting (the reference group for the time pressure variable in model 1.1).

```r
fixef(m1.1_pwr)['time_pressurePlus'] = 0.608
# powerSim(m1.1_pwr, nsim = 1000) #not run
```

Defection vs. time pressure

```r
m1.2 = glmer(behavior_defect ~ time_pressure + round + (1|game) + (1|superid),
             data = exp2data %>% filter(round > 0), family = binomial, nAGQ=0,
             control = glmerControl(optimizer = c("bobyqa"),
                                    optCtrl=list(maxfun=2e5),
                                    calc.derivs=FALSE))

summary(m1.2)
```

```
Generalized linear mixed model fit by maximum likelihood (Adaptive
  Gauss-Hermite Quadrature, nAGQ = 0) [glmerMod]
 Family: binomial  ( logit )
Formula: behavior_defect ~ time_pressure + round + (1 | game) + (1 | superid)
   Data: exp2data %>% filter(round > 0)
Control: glmerControl(optimizer = c("bobyqa"), optCtrl = list(maxfun = 2e+05),
    calc.derivs = FALSE)

     AIC      BIC   logLik deviance df.resid
  5658.6   5695.0  -2824.3   5648.6    10742

Scaled residuals:
    Min      1Q  Median      3Q     Max
```

```
-4.5487 -0.1017  0.0728  0.1006  4.4163

Random effects:
 Groups  Name        Variance Std.Dev.
 superid (Intercept) 35.060   5.921
 game    (Intercept)  3.392   1.842
Number of obs: 10747, groups:  superid, 739; game, 50

Fixed effects:
                   Estimate Std. Error z value Pr(>|z|)
(Intercept)        0.034318   0.502767   0.068    0.946
time_pressurePlus -0.216417   0.703294  -0.308    0.758
round              0.055394   0.009602   5.769 7.97e-09 ***
---
Signif. codes:  0 '***' 0.001 '**' 0.01 '*' 0.05 '.' 0.1 ' ' 1

Correlation of Fixed Effects:
            (Intr) tm_prP
tm_prssrPls -0.699
round       -0.147 -0.002
```

```
# p = 0.758
```

Decision times vs. time pressure (not useful)

```
model_dt_coop = lmer(behaviorTime_sec ~ time_pressure + round + (1|game) + (1|superid),
                     data = exp2data %>% filter(round > 0, behavior_coop == 1))
summary(model_dt_coop)
```

```
Linear mixed model fit by REML. t-tests use Satterthwaite's method [
lmerModLmerTest]
Formula: behaviorTime_sec ~ time_pressure + round + (1 | game) + (1 |
    superid)
   Data: exp2data %>% filter(round > 0, behavior_coop == 1)

REML criterion at convergence: 13885.1

Scaled residuals:
    Min      1Q  Median      3Q     Max
-3.8000 -0.2551 -0.0807  0.1074 21.7822
```

```
Random effects:
 Groups   Name        Variance Std.Dev.
 superid  (Intercept) 1.15374  1.0741
 game     (Intercept) 0.06439  0.2538
 Residual             2.33938  1.5295
Number of obs: 3588, groups:  superid, 432; game, 50

Fixed effects:
                    Estimate Std. Error         df t value Pr(>|t|)
(Intercept)        3.404e+00  1.107e-01  5.194e+01  30.744  < 2e-16 ***
time_pressurePlus -1.275e+00  1.435e-01  3.649e+01  -8.887 1.17e-10 ***
round             -8.243e-03  6.150e-03  3.285e+03  -1.340     0.18
---
Signif. codes:  0 '***' 0.001 '**' 0.01 '*' 0.05 '.' 0.1 ' ' 1

Correlation of Fixed Effects:
            (Intr) tm_prP
tm_prssrPls -0.627
round       -0.423 -0.014
```

```r
model_dt_def = lmer(behaviorTime_sec ~ time_pressure + round + (1|game) + (1|superid),
                    data = exp2data %>% filter(round > 0, behavior_defect == 1))
summary(model_dt_def)
```

```
Linear mixed model fit by REML. t-tests use Satterthwaite's method [
lmerModLmerTest]
Formula: behaviorTime_sec ~ time_pressure + round + (1 | game) + (1 |
    superid)
   Data: exp2data %>% filter(round > 0, behavior_defect == 1)

REML criterion at convergence: 19604.6

Scaled residuals:
    Min      1Q  Median      3Q     Max
-5.6180 -0.2482 -0.0776  0.1050 27.1570

Random effects:
 Groups   Name        Variance Std.Dev.
 superid  (Intercept) 0.945248 0.97224
 game     (Intercept) 0.001146 0.03385
 Residual             2.171540 1.47361
```

```
Number of obs: 5200, groups:  superid, 509; game, 50

Fixed effects:
                    Estimate Std. Error         df t value Pr(>|t|)    
(Intercept)        3.162e+00  7.875e-02  7.030e+01  40.155  < 2e-16 ***
time_pressurePlus -1.055e+00  9.991e-02  4.290e+01 -10.564 1.64e-13 ***
round             -1.729e-02  4.853e-03  4.794e+03  -3.563 0.000371 ***
---
Signif. codes:  0 '***' 0.001 '**' 0.01 '*' 0.05 '.' 0.1 ' ' 1

Correlation of Fixed Effects:
            (Intr) tm_prP
tm_prssrPls -0.593       
round       -0.489 -0.014
```

```r
model_dt_pun = lmer(behaviorTime_sec ~ time_pressure + round + (1|game) + (1|superid),
                    data = exp2data %>% filter(round > 0, behavior_punish == 1))
```

```
boundary (singular) fit: see help('isSingular')
```

```r
summary(model_dt_pun)
```

```
Linear mixed model fit by REML. t-tests use Satterthwaite's method [
lmerModLmerTest]
Formula: behaviorTime_sec ~ time_pressure + round + (1 | game) + (1 |
    superid)
   Data: exp2data %>% filter(round > 0, behavior_punish == 1)

REML criterion at convergence: 2607.7

Scaled residuals: 
    Min      1Q  Median      3Q     Max 
-2.9474 -0.2663 -0.0885  0.0699  9.1708 

Random effects:
 Groups   Name        Variance Std.Dev.
 superid  (Intercept) 3.673    1.917   
 game     (Intercept) 0.000    0.000   
 Residual             6.146    2.479   
Number of obs: 525, groups:  superid, 176; game, 49
```

```
Fixed effects:
                    Estimate Std. Error        df t value Pr(>|t|)
(Intercept)          4.17743    0.32280 283.53251  12.941  < 2e-16 ***
time_pressurePlus   -1.87849    0.39484 127.32898  -4.758 5.23e-06 ***
round               -0.01823    0.02866 450.29088  -0.636    0.525
---
Signif. codes:  0 '***' 0.001 '**' 0.01 '*' 0.05 '.' 0.1 ' ' 1

Correlation of Fixed Effects:
            (Intr) tm_prP
tm_prssrPls -0.437
round       -0.637 -0.077
optimizer (nloptwrap) convergence code: 0 (OK)
boundary (singular) fit: see help('isSingular')
```

Punishment mechanisms vs. time pressure

Punishment for copying/retaliation

```r
m2.1 = glmer(punish_type_CR ~ time_pressure + round + (1|game) + (1|superid),
             data = exp2data %>% filter(round > 0), family = binomial, nAGQ=0,
             control = glmerControl(optimizer = c("bobyqa"),
                                    optCtrl=list(maxfun=2e5),
                                    calc.derivs=FALSE))

summary(m2.1)
```

```
Generalized linear mixed model fit by maximum likelihood (Adaptive
  Gauss-Hermite Quadrature, nAGQ = 0) [glmerMod]
 Family: binomial  ( logit )
Formula: punish_type_CR ~ time_pressure + round + (1 | game) + (1 | superid)
   Data: exp2data %>% filter(round > 0)
Control: glmerControl(optimizer = c("bobyqa"), optCtrl = list(maxfun = 2e+05),
    calc.derivs = FALSE)

     AIC      BIC   logLik deviance df.resid
  1559.8   1596.2   -774.9   1549.8    10742

Scaled residuals:
    Min      1Q  Median      3Q     Max
-1.1200 -0.1048 -0.0669 -0.0467  6.6001
```

```
Random effects:
 Groups  Name        Variance Std.Dev.
 superid (Intercept) 2.722    1.650
 game    (Intercept) 2.117    1.455
Number of obs: 10747, groups:  superid, 739; game, 50

Fixed effects:
                  Estimate Std. Error z value Pr(>|z|)
(Intercept)       -5.15894    0.38747 -13.315   <2e-16 ***
time_pressurePlus -0.07021    0.50659  -0.139     0.89
round              0.02031    0.01840   1.104     0.27
---
Signif. codes:  0 '***' 0.001 '**' 0.01 '*' 0.05 '.' 0.1 ' ' 1

Correlation of Fixed Effects:
            (Intr) tm_prP
tm_prssrPls -0.647
round       -0.392  0.000
```

```
# p = 0.89
```

Punishment for negative reinforcement

```
m2.2 = glmer(punish_type_NR ~ time_pressure + round + (1|game) + (1|superid),
             data = exp2data %>% filter(round > 0), family = binomial, nAGQ=0,
             control = glmerControl(optimizer = c("bobyqa"),
                                    optCtrl=list(maxfun=2e5),
                                    calc.derivs=FALSE))

summary(m2.2)
```

```
Generalized linear mixed model fit by maximum likelihood (Adaptive
  Gauss-Hermite Quadrature, nAGQ = 0) [glmerMod]
 Family: binomial  ( logit )
Formula: punish_type_NR ~ time_pressure + round + (1 | game) + (1 | superid)
   Data: exp2data %>% filter(round > 0)
Control: glmerControl(optimizer = c("bobyqa"), optCtrl = list(maxfun = 2e+05),
    calc.derivs = FALSE)

     AIC      BIC   logLik deviance df.resid
```

```
  2747.8   2784.2  -1368.9   2737.8    10742

Scaled residuals:
    Min      1Q  Median      3Q     Max
-1.5280 -0.1039 -0.0845 -0.0722  5.0321

Random effects:
 Groups   Name        Variance Std.Dev.
 superid (Intercept) 4.903    2.214
 game    (Intercept) 1.007    1.004
Number of obs: 10747, groups:  superid, 739; game, 50

Fixed effects:
                  Estimate Std. Error z value Pr(>|z|)
(Intercept)       -4.55764    0.29037 -15.696   <2e-16 ***
time_pressurePlus -0.18463    0.38230  -0.483   0.6291
round              0.03579    0.01325   2.702   0.0069 **
---
Signif. codes:  0 '***' 0.001 '**' 0.01 '*' 0.05 '.' 0.1 ' ' 1

Correlation of Fixed Effects:
            (Intr) tm_prP
tm_prssrPls -0.647
round       -0.384  0.000
```

```
# p = 0.629
```

Punishment for inequality aversion

```
m2.3 = glmer(punish_type_IA ~ time_pressure + round + (1|game) + (1|superid),
             data = exp2data %>% filter(round > 0), family = binomial, nAGQ=0,
             control = glmerControl(optimizer = c("bobyqa"),
                                    optCtrl=list(maxfun=2e5),
                                    calc.derivs=FALSE))
summary(m2.3)
```

```
Generalized linear mixed model fit by maximum likelihood (Adaptive
  Gauss-Hermite Quadrature, nAGQ = 0) [glmerMod]
 Family: binomial  ( logit )
Formula: punish_type_IA ~ time_pressure + round + (1 | game) + (1 | superid)
   Data: exp2data %>% filter(round > 0)
```

```
Control: glmerControl(optimizer = c("bobyqa"), optCtrl = list(maxfun = 2e+05),
    calc.derivs = FALSE)

     AIC      BIC   logLik deviance df.resid
  2697.2   2733.6  -1343.6   2687.2    10742

Scaled residuals:
    Min      1Q  Median      3Q     Max
-1.3832 -0.0838 -0.0767 -0.0709  4.6221

Random effects:
 Groups  Name        Variance Std.Dev.
 superid (Intercept) 6.9558   2.6374
 game    (Intercept) 0.4017   0.6338
Number of obs: 10747, groups:  superid, 739; game, 50

Fixed effects:
                  Estimate Std. Error z value Pr(>|z|)
(Intercept)       -4.56425    0.25976 -17.571   <2e-16 ***
time_pressurePlus -0.26515    0.33947  -0.781   0.4348
round              0.02203    0.01332   1.654   0.0981 .
---
Signif. codes:  0 '***' 0.001 '**' 0.01 '*' 0.05 '.' 0.1 ' ' 1

Correlation of Fixed Effects:
            (Intr) tm_prP
tm_prssrPls -0.630
round       -0.420 -0.001
```

```
# p = 0.435
```

Unclassified punishment

```
m2.4 = glmer(punish_type_U ~ time_pressure + round + (1|game) + (1|superid),
             data = exp2data %>% filter(round > 0), family = binomial, nAGQ=0,
             control = glmerControl(optimizer = c("bobyqa"),
                                    optCtrl=list(maxfun=2e5),
                                    calc.derivs=FALSE))
summary(m2.4)
```

```
Generalized linear mixed model fit by maximum likelihood (Adaptive
```

```
  Gauss-Hermite Quadrature, nAGQ = 0) [glmerMod]
 Family: binomial  ( logit )
Formula: punish_type_U ~ time_pressure + round + (1 | game) + (1 | superid)
   Data: exp2data %>% filter(round > 0)
Control: glmerControl(optimizer = c("bobyqa"), optCtrl = list(maxfun = 2e+05),
    calc.derivs = FALSE)

     AIC      BIC   logLik deviance df.resid
  1220.2   1256.6   -605.1   1210.2    10742

Scaled residuals:
    Min      1Q  Median      3Q     Max
-1.4674 -0.0904 -0.0623 -0.0419 11.0773

Random effects:
 Groups  Name        Variance Std.Dev.
 superid (Intercept) 3.919    1.9798
 game    (Intercept) 0.358    0.5984
Number of obs: 10747, groups:  superid, 739; game, 50

Fixed effects:
                  Estimate Std. Error z value Pr(>|z|)
(Intercept)       -3.86428    0.26454 -14.607  < 2e-16 ***
time_pressurePlus -0.15285    0.33542  -0.456    0.649
round             -0.18400    0.02469  -7.452  9.2e-14 ***
---
Signif. codes:  0 '***' 0.001 '**' 0.01 '*' 0.05 '.' 0.1 ' ' 1

Correlation of Fixed Effects:
            (Intr) tm_prP
tm_prssrPls -0.614
round       -0.471  0.000
```

```
# p = 0.649
```

Decision time as the outcome

Exp 1 - Cooperation as reference

```
m4 = lmer(behaviorTime_sec ~ behavior + round + (1|game) + (1|superid),
          data = exp1data %>%
```

```r
            filter(round > 0))

summary(m4)
```

```
Linear mixed model fit by REML. t-tests use Satterthwaite's method [
lmerModLmerTest]
Formula: behaviorTime_sec ~ behavior + round + (1 | game) + (1 | superid)
   Data: exp1data %>% filter(round > 0)

REML criterion at convergence: 69452.7

Scaled residuals:
    Min      1Q  Median      3Q     Max 
-2.9109 -0.3656 -0.1566  0.0373 10.4105 

Random effects:
 Groups   Name        Variance Std.Dev.
 superid  (Intercept) 11.789   3.434   
 game     (Intercept)  1.824   1.350   
 Residual             64.809   8.050   
Number of obs: 9776, groups:  superid, 719; game, 50

Fixed effects:
              Estimate Std. Error         df t value Pr(>|t|)    
(Intercept)    6.79184    0.30462  110.40898  22.296  < 2e-16 ***
behaviorD      0.30411    0.23036 5471.24286   1.320  0.18684    
behaviorP      1.21569    0.42149 9059.09346   2.884  0.00393 ** 
round         -0.16708    0.01916 9287.78481  -8.719  < 2e-16 ***
---
Signif. codes:  0 '***' 0.001 '**' 0.01 '*' 0.05 '.' 0.1 ' ' 1

Correlation of Fixed Effects:
          (Intr) behvrD behvrP
behaviorD -0.322              
behaviorP -0.180  0.263       
round     -0.460 -0.058  0.023
```

Exp 1 - Defection as reference

```r
m4_1 = lmer(behaviorTime_sec ~ factor(behavior, levels = c("D", "C", "P")) +
              round + (1|game) + (1|superid), data = exp1data)
summary(m4_1)
```

```
Linear mixed model fit by REML. t-tests use Satterthwaite's method [
lmerModLmerTest]
Formula: behaviorTime_sec ~ factor(behavior, levels = c("D", "C", "P")) +
    round + (1 | game) + (1 | superid)
   Data: exp1data

REML criterion at convergence: 69452.7

Scaled residuals:
    Min      1Q  Median      3Q     Max
-2.9109 -0.3656 -0.1566  0.0373 10.4105

Random effects:
 Groups   Name        Variance Std.Dev.
 superid  (Intercept) 11.789   3.434
 game     (Intercept)  1.824   1.350
 Residual             64.809   8.050
Number of obs: 9776, groups:  superid, 719; game, 50

Fixed effects:
                                              Estimate Std. Error         df
(Intercept)                                    7.09595    0.31735  125.30780
factor(behavior, levels = c("D", "C", "P"))C  -0.30411    0.23036 5471.24286
factor(behavior, levels = c("D", "C", "P"))P   0.91157    0.42392 8963.54750
round                                         -0.16708    0.01916 9287.78482
                                             t value Pr(>|t|)
(Intercept)                                   22.360   <2e-16 ***
factor(behavior, levels = c("D", "C", "P"))C  -1.320   0.1868
factor(behavior, levels = c("D", "C", "P"))P   2.150   0.0316 *
round                                         -8.719   <2e-16 ***
---
Signif. codes:  0 '***' 0.001 '**' 0.01 '*' 0.05 '.' 0.1 ' ' 1

Correlation of Fixed Effects:
                      (Intr) f(,l=c("D","C","P"))C f(,l=c("D","C","P"))P
f(,l=c("D","C","P"))C -0.417
f(,l=c("D","C","P"))P -0.209  0.282
round                 -0.484  0.058                 0.054
```

Exp 1 - Comparing the punishment mechanisms - CR punishment is the reference

```
m4_2 = lmer(behaviorTime_sec ~ punish_type_NR + punish_type_IA + punish_type_U +
              round + (1|game) + (1|superid),
            data = exp1data %>% filter(behavior_punish == 1))
summary(m4_2)
```

```
Linear mixed model fit by REML. t-tests use Satterthwaite's method [
lmerModLmerTest]
Formula: behaviorTime_sec ~ punish_type_NR + punish_type_IA + punish_type_U +
    round + (1 | game) + (1 | superid)
   Data: exp1data %>% filter(behavior_punish == 1)

REML criterion at convergence: 3686.3

Scaled residuals:
    Min      1Q  Median      3Q     Max
-1.7665 -0.4121 -0.1780  0.0855  6.0832

Random effects:
 Groups   Name        Variance Std.Dev.
 superid  (Intercept) 23.47    4.845
 game     (Intercept) 11.29    3.360
 Residual             64.44    8.028
Number of obs: 508, groups:  superid, 174; game, 48

Fixed effects:
               Estimate Std. Error       df t value Pr(>|t|)
(Intercept)      6.2481     1.5349 329.3702   4.071 5.88e-05 ***
punish_type_NR   0.5307     1.1866 487.8494   0.447   0.6549
punish_type_IA   1.5698     1.1200 424.9994   1.402   0.1618
punish_type_U    3.7815     1.6083 494.5013   2.351   0.0191 *
round           -0.1209     0.1023 479.7367  -1.182   0.2377
---
Signif. codes:  0 '***' 0.001 '**' 0.01 '*' 0.05 '.' 0.1 ' ' 1

Correlation of Fixed Effects:
            (Intr) pn__NR pn__IA pns__U
pnsh_typ_NR -0.485
pnsh_typ_IA -0.531  0.084
punsh_typ_U -0.608  0.501  0.464
round       -0.364 -0.197 -0.061 -0.070
```

Exp 1 - Comparing copying/retaliation punishment vs. all others

```
m4_2_1 = lmer(behaviorTime_sec ~ punish_type_CR + round + (1|game) + (1|superid),
              data = exp1data %>%
  filter(behavior_punish == 1))
summary(m4_2_1)
```

```
Linear mixed model fit by REML. t-tests use Satterthwaite's method [
lmerModLmerTest]
Formula: behaviorTime_sec ~ punish_type_CR + round + (1 | game) + (1 |
    superid)
   Data: exp1data %>% filter(behavior_punish == 1)

REML criterion at convergence: 3690.5

Scaled residuals:
    Min      1Q  Median      3Q     Max
-1.7020 -0.4204 -0.1869  0.0978  6.1235

Random effects:
 Groups   Name        Variance Std.Dev.
 superid  (Intercept) 23.89    4.887
 game     (Intercept) 10.43    3.229
 Residual             64.10    8.006
Number of obs: 508, groups:  superid, 174; game, 48

Fixed effects:
               Estimate Std. Error       df t value Pr(>|t|)
(Intercept)      9.1691     1.1074 169.5893   8.280 3.54e-14 ***
punish_type_CR  -2.4355     0.9306 483.1002  -2.617  0.00914 **
round           -0.1489     0.1003 479.6408  -1.484  0.13842
---
Signif. codes:  0 '***' 0.001 '**' 0.01 '*' 0.05 '.' 0.1 ' ' 1

Correlation of Fixed Effects:
            (Intr) pn__CR
pnsh_typ_CR -0.308
round       -0.693  0.113
```

Exp 1 - Comparing unclassified punishment vs. all others

```r
m4_2_2 = lmer(behaviorTime_sec ~ punish_type_U + round + (1|game) + (1|superid),
              data = exp1data %>%
  filter(behavior_punish == 1))
summary(m4_2_2)
```

```
Linear mixed model fit by REML. t-tests use Satterthwaite's method [
lmerModLmerTest]
Formula: behaviorTime_sec ~ punish_type_U + round + (1 | game) + (1 |
    superid)
   Data: exp1data %>% filter(behavior_punish == 1)

REML criterion at convergence: 4089.8

Scaled residuals:
    Min      1Q  Median      3Q     Max
-1.9010 -0.4359 -0.1943  0.0883  6.0749

Random effects:
 Groups   Name        Variance Std.Dev.
 superid  (Intercept) 24.642   4.964
 game     (Intercept)  8.374   2.894
 Residual             67.666   8.226
Number of obs: 560, groups:  superid, 184; game, 49

Fixed effects:
               Estimate Std. Error        df t value Pr(>|t|)
(Intercept)     7.80336    1.06843 157.29632   7.304 1.32e-11 ***
punish_type_U   2.10507    1.02123 549.35836   2.061   0.0397 *
round          -0.09103    0.09612 538.05539  -0.947   0.3440
---
Signif. codes:  0 '***' 0.001 '**' 0.01 '*' 0.05 '.' 0.1 ' ' 1

Correlation of Fixed Effects:
            (Intr) pns__U
punsh_typ_U -0.464
round       -0.695  0.304
```

Exp 1 - Regression model with and without punishment in round t-1

```r
m13 = lmer(behaviorTime_sec ~ round + last_punished + (1|game) + (1|superid),
           data = exp1data %>% filter(behavior_punish == 1))
```

```r
summary(m13)
```

```
Linear mixed model fit by REML. t-tests use Satterthwaite's method [
lmerModLmerTest]
Formula: behaviorTime_sec ~ round + last_punished + (1 | game) + (1 |
    superid)
   Data: exp1data %>% filter(behavior_punish == 1)

REML criterion at convergence: 3690.5

Scaled residuals:
    Min      1Q  Median      3Q     Max
-1.7020 -0.4204 -0.1869  0.0978  6.1235

Random effects:
 Groups   Name        Variance Std.Dev.
 superid  (Intercept) 23.89    4.887
 game     (Intercept) 10.43    3.229
 Residual             64.10    8.006
Number of obs: 508, groups:  superid, 174; game, 48

Fixed effects:
              Estimate Std. Error       df t value Pr(>|t|)
(Intercept)     9.1691     1.1074 169.5893   8.280 3.54e-14 ***
round          -0.1489     0.1003 479.6408  -1.484  0.13842
last_punished  -2.4355     0.9306 483.1002  -2.617  0.00914 **
---
Signif. codes:  0 '***' 0.001 '**' 0.01 '*' 0.05 '.' 0.1 ' ' 1

Correlation of Fixed Effects:
            (Intr) round
round       -0.693
last_punshd -0.308  0.113
```

Exp 2 - Controlling for time pressure

Time pressure +

```r
exp2_tp_plus = exp2data %>% filter(time_pressure == "Plus")
exp2_tp_minus = exp2data %>% filter(time_pressure == "Minus")

m5a = lmer(behaviorTime_sec ~ behavior + round + (1|game) + (1|superid),
           data = exp2_tp_plus %>% filter(round > 0))
summary(m5a)
```

```
Linear mixed model fit by REML. t-tests use Satterthwaite's method [
lmerModLmerTest]
Formula: behaviorTime_sec ~ behavior + round + (1 | game) + (1 | superid)
   Data: exp2_tp_plus %>% filter(round > 0)

REML criterion at convergence: 2356.9

Scaled residuals:
    Min      1Q  Median      3Q     Max
-3.2207 -0.6608 -0.0951  0.5662  4.8321

Random effects:
 Groups   Name        Variance Std.Dev.
 superid  (Intercept) 0.07370  0.27147
 game     (Intercept) 0.00315  0.05613
 Residual             0.08420  0.29018
Number of obs: 4066, groups:  superid, 367; game, 25

Fixed effects:
              Estimate Std. Error         df t value Pr(>|t|)
(Intercept)  2.090e+00  2.348e-02  6.115e+01  88.999  < 2e-16 ***
behaviorD   -5.140e-02  1.966e-02  2.483e+03  -2.614  0.00899 **
behaviorP    1.155e-01  2.799e-02  4.043e+03   4.128 3.73e-05 ***
round       -6.124e-03  1.091e-03  3.751e+03  -5.611 2.15e-08 ***
---
Signif. codes:  0 '***' 0.001 '**' 0.01 '*' 0.05 '.' 0.1 ' ' 1

Correlation of Fixed Effects:
          (Intr) behvrD behvrP
behaviorD -0.456
behaviorP -0.206  0.315
round     -0.358 -0.043 -0.001
```

Time pressure -

```r
m5b = lmer(behaviorTime_sec ~ behavior + round + (1|game) + (1|superid),
           data = exp2_tp_minus %>% filter(round > 0))

summary(m5b)
```

```
Linear mixed model fit by REML. t-tests use Satterthwaite's method [
lmerModLmerTest]
Formula: behaviorTime_sec ~ behavior + round + (1 | game) + (1 | superid)
   Data: exp2_tp_minus %>% filter(round > 0)

REML criterion at convergence: 23330.5

Scaled residuals:
    Min      1Q  Median      3Q     Max
-3.7670 -0.2960 -0.1224  0.0783 19.2939

Random effects:
 Groups   Name        Variance Std.Dev.
 superid  (Intercept) 1.58559  1.2592
 game     (Intercept) 0.08172  0.2859
 Residual             4.38568  2.0942
Number of obs: 5247, groups:  superid, 366; game, 25

Fixed effects:
              Estimate Std. Error        df t value Pr(>|t|)
(Intercept)  3.330e+00  1.228e-01  6.772e+01  27.117  < 2e-16 ***
behaviorD   -2.740e-01  1.069e-01  2.005e+03  -2.562  0.01048 *
behaviorP    4.700e-01  1.475e-01  5.177e+03   3.185  0.00145 **
round       -2.073e-02  6.745e-03  4.913e+03  -3.074  0.00213 **
---
Signif. codes:  0 '***' 0.001 '**' 0.01 '*' 0.05 '.' 0.1 ' ' 1

Correlation of Fixed Effects:
          (Intr) behvrD behvrP
behaviorD -0.491
behaviorP -0.253  0.357
round     -0.412 -0.037  0.009
```

Supplementary Analyses for Verification

Testing interaction between punishing environment and punishment decision making (Table S6)

```
exp1data = exp1data %>%
  mutate(local_rate_punish_lag_binary =
           case_when(local_rate_punish_lag == 0 ~ 'Low',
                     local_rate_punish_lag != 0 ~ 'High'))

m4_no_int = lmer(behaviorTime_sec ~ behavior_punish + local_rate_punish_lag +
                  round + (1|game) + (1|superid),
                data = exp1data %>% filter(round > 1))

m4_int = lmer(behaviorTime_sec ~ behavior_punish*local_rate_punish_lag +
                  round + (1|game) + (1|superid),
                data = exp1data %>% filter(round > 1))

summary(m4_int)
```

```
Linear mixed model fit by REML. t-tests use Satterthwaite's method [
lmerModLmerTest]
Formula: behaviorTime_sec ~ behavior_punish * local_rate_punish_lag +
    round + (1 | game) + (1 | superid)
   Data: exp1data %>% filter(round > 1)

REML criterion at convergence: 63408.5

Scaled residuals:
    Min      1Q  Median      3Q     Max
-3.0493 -0.3578 -0.1520  0.0321 10.7635

Random effects:
 Groups   Name        Variance Std.Dev.
 superid  (Intercept) 11.076   3.328
 game     (Intercept)  1.554   1.247
 Residual             59.983   7.745
Number of obs: 9020, groups:  superid, 713; game, 50

Fixed effects:
                                       Estimate Std. Error        df t value
(Intercept)                              6.5745     0.2951  116.7952  22.276
```

```
behavior_punish                              1.2756     0.4545 8494.5666   2.806
local_rate_punish_lag                       -0.6620     0.8331 8724.9751  -0.795
round                                       -0.1310     0.0206 8489.2586  -6.360
behavior_punish:local_rate_punish_lag       -4.0876     2.4373 8772.9935  -1.677
                                     Pr(>|t|)
(Intercept)                           < 2e-16 ***
behavior_punish                       0.00502 **
local_rate_punish_lag                 0.42684
round                                2.12e-10 ***
behavior_punish:local_rate_punish_lag 0.09356 .
---
Signif. codes:  0 '***' 0.001 '**' 0.01 '*' 0.05 '.' 0.1 ' ' 1

Correlation of Fixed Effects:
            (Intr) bhvr_p lcl___ round
behavr_pnsh -0.114
lcl_rt_pns_ -0.196  0.102
round       -0.584  0.041  0.086
bhvr_pn:___  0.059 -0.418 -0.309 -0.019
```

Regression analysis for the effect of low initial wealth allocation on decision time (Tables S7, S8)

```
summary(lmer(behaviorTime_sec ~ initial_score_low + behavior + round + (1|game)
             + (1|superid), data = exp1data))
```

```
Linear mixed model fit by REML. t-tests use Satterthwaite's method [
lmerModLmerTest]
Formula: behaviorTime_sec ~ initial_score_low + behavior + round + (1 |
    game) + (1 | superid)
   Data: exp1data

REML criterion at convergence: 69452.7

Scaled residuals:
    Min      1Q  Median      3Q     Max
-2.9146 -0.3656 -0.1562  0.0382 10.4141

Random effects:
 Groups   Name        Variance Std.Dev.
 superid  (Intercept) 11.806   3.436
```

```
 game     (Intercept)  1.826   1.351  
 Residual             64.809   8.050  
Number of obs: 9776, groups:  superid, 719; game, 50

Fixed effects:
                    Estimate Std. Error         df t value Pr(>|t|)    
(Intercept)          6.66573    0.38930  253.32765  17.122  < 2e-16 ***
initial_score_low    0.17879    0.34334  603.94338   0.521  0.60275    
behaviorD            0.30663    0.23045 5479.92156   1.331  0.18339    
behaviorP            1.21876    0.42156 9060.96987   2.891  0.00385 ** 
round               -0.16710    0.01916 9287.58879  -8.719  < 2e-16 ***
---
Signif. codes:  0 '***' 0.001 '**' 0.01 '*' 0.05 '.' 0.1 ' ' 1

Correlation of Fixed Effects:
            (Intr) intl__ behvrD behvrP
intl_scr_lw -0.622                     
behaviorD   -0.265  0.022              
behaviorP   -0.150  0.015  0.263       
round       -0.358 -0.003 -0.058  0.023
```

```r
summary(lmer(behaviorTime_sec ~ initial_score_low + behavior + round +
               (1|game) + (1|superid),
             data = exp2data %>% filter(time_pressure == 'Minus')))
```

```
Linear mixed model fit by REML. t-tests use Satterthwaite's method [
lmerModLmerTest]
Formula: behaviorTime_sec ~ initial_score_low + behavior + round + (1 |  
    game) + (1 | superid)
   Data: exp2data %>% filter(time_pressure == "Minus")

REML criterion at convergence: 23332

Scaled residuals: 
    Min      1Q  Median      3Q     Max 
-3.7773 -0.2968 -0.1225  0.0778 19.2842 

Random effects:
 Groups   Name        Variance Std.Dev.
 superid  (Intercept) 1.58849  1.2604  
 game     (Intercept) 0.08541  0.2922  
 Residual             4.38547  2.0942  
```

```
Number of obs: 5247, groups:  superid, 366; game, 25

Fixed effects:
                    Estimate Std. Error         df t value Pr(>|t|)
(Intercept)        3.272e+00  1.640e-01  1.616e+02  19.952  < 2e-16 ***
initial_score_low  8.524e-02  1.583e-01  3.379e+02   0.538  0.59060
behaviorD         -2.749e-01  1.070e-01  2.007e+03  -2.570  0.01025 *
behaviorP          4.687e-01  1.476e-01  5.176e+03   3.176  0.00150 **
round             -2.072e-02  6.745e-03  4.913e+03  -3.072  0.00214 **
---
Signif. codes:  0 '***' 0.001 '**' 0.01 '*' 0.05 '.' 0.1 ' ' 1

Correlation of Fixed Effects:
            (Intr) intl__ behvrD behvrP
intl_scr_lw -0.658
behaviorD   -0.363 -0.008
behaviorP   -0.182 -0.011  0.357
round       -0.310  0.003 -0.037  0.009
```

```
summary(lmer(behaviorTime_sec ~ behavior + initial_score_low + round + (1|game)
             + (1|superid), data = exp2data %>% filter(time_pressure == 'Plus')))
```

```
Linear mixed model fit by REML. t-tests use Satterthwaite's method [
lmerModLmerTest]
Formula: behaviorTime_sec ~ behavior + initial_score_low + round + (1 |
    game) + (1 | superid)
   Data: exp2data %>% filter(time_pressure == "Plus")

REML criterion at convergence: 2361.6

Scaled residuals:
    Min      1Q  Median      3Q     Max
-3.2220 -0.6618 -0.0931  0.5661  4.8309

Random effects:
 Groups   Name        Variance Std.Dev.
 superid  (Intercept) 0.073843 0.27174
 game     (Intercept) 0.003195 0.05653
 Residual             0.084201 0.29017
Number of obs: 4066, groups:  superid, 367; game, 25

Fixed effects:
```

```
                     Estimate Std. Error        df t value Pr(>|t|)
(Intercept)         2.076e+00  3.285e-02 1.733e+02  63.197  < 2e-16 ***
behaviorD          -5.128e-02  1.967e-02 2.484e+03  -2.607  0.00918 **
behaviorP           1.158e-01  2.799e-02 4.041e+03   4.138 3.58e-05 ***
initial_score_low   2.027e-02  3.296e-02 3.489e+02   0.615  0.53909
round              -6.126e-03  1.091e-03 3.751e+03  -5.613 2.13e-08 ***
---
Signif. codes:  0 '***' 0.001 '**' 0.01 '*' 0.05 '.' 0.1 ' ' 1

Correlation of Fixed Effects:
            (Intr) behvrD behvrP intl__
behaviorD   -0.330
behaviorP   -0.158  0.315
intl_scr_lw -0.698  0.005  0.016
round       -0.255 -0.043 -0.001 -0.001
```

```
summary(lmer(behaviorTime_sec ~ punish_type_NR + punish_type_IA + punish_type_U
             + initial_score_low +
              round + (1|game) + (1|superid),
            data = exp1data %>% filter(behavior_punish == 1)))
```

```
Linear mixed model fit by REML. t-tests use Satterthwaite's method [
lmerModLmerTest]
Formula: behaviorTime_sec ~ punish_type_NR + punish_type_IA + punish_type_U +
    initial_score_low + round + (1 | game) + (1 | superid)
   Data: exp1data %>% filter(behavior_punish == 1)

REML criterion at convergence: 3683.3

Scaled residuals:
    Min      1Q  Median      3Q     Max
-1.8090 -0.4179 -0.1820  0.0792  6.0808

Random effects:
 Groups   Name        Variance Std.Dev.
 superid  (Intercept) 24.29    4.928
 game     (Intercept) 11.12    3.335
 Residual             64.20    8.013
Number of obs: 508, groups:  superid, 174; game, 48

Fixed effects:
                   Estimate Std. Error       df t value Pr(>|t|)
```

```
(Intercept)          5.7683     1.6748 290.7348   3.444 0.000657 ***
punish_type_NR       0.5899     1.1890 484.0219   0.496 0.620008
punish_type_IA       1.2323     1.2123 469.3862   1.017 0.309910
punish_type_U        3.7892     1.6085 493.0342   2.356 0.018876 *
initial_score_low    1.0097     1.3814 120.4577   0.731 0.466256
round               -0.1250     0.1024 478.1856  -1.221 0.222565
---
Signif. codes:  0 '***' 0.001 '**' 0.01 '*' 0.05 '.' 0.1 ' ' 1

Correlation of Fixed Effects:
            (Intr) pn__NR pn__IA pns__U intl__
pnsh_typ_NR -0.469
pnsh_typ_IA -0.299  0.053
punsh_typ_U -0.555  0.499  0.431
intl_scr_lw -0.399  0.064 -0.379 -0.006
round       -0.315 -0.200 -0.039 -0.070 -0.046
```

```r
summary(lmer(behaviorTime_sec ~ punish_type_NR + punish_type_IA + punish_type_U
             + initial_score_low +
              round + (1|game) + (1|superid),
            data = exp2data %>%
              filter(behavior_punish == 1, time_pressure == 'Minus')))
```

```
boundary (singular) fit: see help('isSingular')

Linear mixed model fit by REML. t-tests use Satterthwaite's method [
lmerModLmerTest]
Formula: behaviorTime_sec ~ punish_type_NR + punish_type_IA + punish_type_U +
    initial_score_low + round + (1 | game) + (1 | superid)
   Data: exp2data %>% filter(behavior_punish == 1, time_pressure == "Minus")

REML criterion at convergence: 1826.1

Scaled residuals:
    Min      1Q  Median      3Q     Max
-2.5741 -0.2987 -0.1420  0.0779  7.4304

Random effects:
 Groups   Name        Variance Std.Dev.
 superid  (Intercept) 6.570    2.563
 game     (Intercept) 0.000    0.000
 Residual             9.037    3.006
```

```
Number of obs: 341, groups:  superid, 100; game, 25

Fixed effects:
                    Estimate Std. Error        df t value Pr(>|t|)    
(Intercept)          4.10574    0.88082 190.10165   4.661  5.9e-06 ***
punish_type_NR       1.03461    0.50453 307.49181   2.051   0.0411 *  
punish_type_IA      -0.14245    0.59177 334.70019  -0.241   0.8099    
punish_type_U        0.37886    0.71566 278.32617   0.529   0.5970    
initial_score_low   -0.57891    0.73529  59.19624  -0.787   0.4342    
round               -0.03548    0.04477 297.16389  -0.792   0.4287    
---
Signif. codes:  0 '***' 0.001 '**' 0.01 '*' 0.05 '.' 0.1 ' ' 1

Correlation of Fixed Effects:
            (Intr) pn__NR pn__IA pns__U intl__
pnsh_typ_NR -0.422                            
pnsh_typ_IA -0.398  0.049                     
punsh_typ_U -0.593  0.467  0.574              
intl_scr_lw -0.568  0.018 -0.140  0.011       
round       -0.299 -0.107 -0.127  0.046  0.032
optimizer (nloptwrap) convergence code: 0 (OK)
boundary (singular) fit: see help('isSingular')
```

```
summary(lmer(behaviorTime_sec ~ punish_type_NR + punish_type_IA + punish_type_U
             + initial_score_low +
              round + (1|game) + (1|superid),
            data = exp2data %>%
              filter(behavior_punish == 1, time_pressure == 'Plus')))
```

```
Linear mixed model fit by REML. t-tests use Satterthwaite's method [
lmerModLmerTest]
Formula: behaviorTime_sec ~ punish_type_NR + punish_type_IA + punish_type_U +
    initial_score_low + round + (1 | game) + (1 | superid)
   Data: exp2data %>% filter(behavior_punish == 1, time_pressure == "Plus")

REML criterion at convergence: 174.3

Scaled residuals: 
     Min       1Q   Median       3Q      Max 
-1.83298 -0.55507  0.02874  0.60241  2.32159 

Random effects:
```

```
 Groups   Name        Variance Std.Dev.
 superid  (Intercept) 0.11062  0.33260
 game     (Intercept) 0.00683  0.08265
 Residual             0.07639  0.27638
Number of obs: 184, groups:  superid, 76; game, 24

Fixed effects:
                    Estimate Std. Error         df t value Pr(>|t|)
(Intercept)         2.185287   0.116628 124.848840  18.737   <2e-16 ***
punish_type_NR      0.024358   0.068415 146.332603   0.356    0.722
punish_type_IA     -0.025223   0.074085 155.102352  -0.340    0.734
punish_type_U      -0.001092   0.097554 137.910560  -0.011    0.991
initial_score_low   0.019143   0.102787  74.808561   0.186    0.853
round              -0.004240   0.006033 126.423327  -0.703    0.483
---
Signif. codes:  0 '***' 0.001 '**' 0.01 '*' 0.05 '.' 0.1 ' ' 1

Correlation of Fixed Effects:
            (Intr) pn__NR pn__IA pns__U intl__
pnsh_typ_NR -0.442
pnsh_typ_IA -0.303  0.103
punsh_typ_U -0.532  0.558  0.451
intl_scr_lw -0.464 -0.072 -0.296 -0.088
round       -0.457 -0.035 -0.053  0.156  0.087
```

Evaluating effect of past round defection rate on inequality aversion punishment (Table S9)

```
IA_data_e1 = exp1data %>% filter(punish_type_IA == T) %>%
  mutate(high_defect_rate_lag = ifelse(local_rate_defect_lag > 0.5,
                                        'Defect rate > 0.5', "Defect rate <= 0.5"))

summary(lmer(behaviorTime_sec ~ high_defect_rate_lag + round + (1|game) +
               (1|superid), data = IA_data_e1))
```

```
boundary (singular) fit: see help('isSingular')
```

```
Linear mixed model fit by REML. t-tests use Satterthwaite's method [
lmerModLmerTest]
Formula: behaviorTime_sec ~ high_defect_rate_lag + round + (1 | game) +
    (1 | superid)
```

```
   Data: IA_data_e1

REML criterion at convergence: 2039

Scaled residuals: 
    Min      1Q  Median      3Q     Max 
-2.1463 -0.4384 -0.2147  0.0979  5.9454 

Random effects:
 Groups   Name        Variance Std.Dev.
 superid  (Intercept)  0.00    0.000   
 game     (Intercept) 29.43    5.425   
 Residual             66.96    8.183   
Number of obs: 283, groups:  superid, 119; game, 43

Fixed effects:
                                       Estimate Std. Error        df t value
(Intercept)                             7.00558    1.52616 128.04269   4.590
high_defect_rate_lagDefect rate > 0.5   1.76945    1.28524 276.37411   1.377
round                                  -0.07426    0.14125 278.96536  -0.526
                                      Pr(>|t|)    
(Intercept)                           1.04e-05 ***
high_defect_rate_lagDefect rate > 0.5    0.170    
round                                    0.599    
---
Signif. codes:  0 '***' 0.001 '**' 0.01 '*' 0.05 '.' 0.1 ' ' 1

Correlation of Fixed Effects:
            (Intr) h__r>0
hg___Dr>0.5 -0.272       
round       -0.577 -0.291
optimizer (nloptwrap) convergence code: 0 (OK)
boundary (singular) fit: see help('isSingular')
```

```
IA_data_e2 = exp2data %>%
  filter(punish_type_IA == T) %>%
  mutate(high_defect_rate_lag = ifelse(local_rate_defect_lag > 0.5,
                                       'Defect rate > 0.5',
                                       "Defect rate <= 0.5"))

summary(lmer(behaviorTime_sec ~ high_defect_rate_lag + round + (1|game) +
               (1|superid),
```

```
               data = IA_data_e2 %>% filter(time_pressure == 'Minus')))
```

```
Linear mixed model fit by REML. t-tests use Satterthwaite's method [
lmerModLmerTest]
Formula: behaviorTime_sec ~ high_defect_rate_lag + round + (1 | game) +
    (1 | superid)
   Data: IA_data_e2 %>% filter(time_pressure == "Minus")

REML criterion at convergence: 1192.2

Scaled residuals:
    Min      1Q  Median      3Q     Max
-2.0322 -0.3084 -0.1641  0.0425  6.4899

Random effects:
 Groups   Name        Variance  Std.Dev.
 superid  (Intercept)  6.414773 2.53274
 game     (Intercept)  0.009413 0.09702
 Residual             12.457652 3.52954
Number of obs: 211, groups:  superid, 75; game, 25

Fixed effects:
                                       Estimate Std. Error        df t value
(Intercept)                             3.52903    0.74703 149.44054   4.724
high_defect_rate_lagDefect rate > 0.5   1.25054    0.59541 188.67712   2.100
round                                  -0.03702    0.07108 183.67433  -0.521
                                      Pr(>|t|)
(Intercept)                           5.28e-06 ***
high_defect_rate_lagDefect rate > 0.5    0.037 *
round                                    0.603
---
Signif. codes:  0 '***' 0.001 '**' 0.01 '*' 0.05 '.' 0.1 ' ' 1

Correlation of Fixed Effects:
            (Intr) h__r>0
hg___Dr>0.5 -0.407
round       -0.662 -0.162
```

```
summary(lmer(behaviorTime_sec ~ high_defect_rate_lag +
               round + (1|game) + (1|superid),
             data = IA_data_e2 %>% filter(time_pressure == 'Plus')))
```

```
boundary (singular) fit: see help('isSingular')


Linear mixed model fit by REML. t-tests use Satterthwaite's method [
lmerModLmerTest]
Formula: behaviorTime_sec ~ high_defect_rate_lag + round + (1 | game) +
    (1 | superid)
   Data: IA_data_e2 %>% filter(time_pressure == "Plus")

REML criterion at convergence: 109

Scaled residuals:
     Min       1Q   Median       3Q      Max
-1.71800 -0.55936  0.07293  0.51427  2.17703

Random effects:
 Groups   Name        Variance Std.Dev.
 superid  (Intercept) 0.12424  0.3525
 game     (Intercept) 0.00000  0.0000
 Residual             0.06913  0.2629
Number of obs: 113, groups:  superid, 55; game, 22

Fixed effects:
                                        Estimate Std. Error         df t value
(Intercept)                             2.116992   0.098005 109.456692  21.601
high_defect_rate_lagDefect rate > 0.5   0.095327   0.066173  85.348506   1.441
round                                  -0.002140   0.008578  83.358857  -0.249
                                      Pr(>|t|)
(Intercept)                             <2e-16 ***
high_defect_rate_lagDefect rate > 0.5    0.153
round                                    0.804
---
Signif. codes:  0 '***' 0.001 '**' 0.01 '*' 0.05 '.' 0.1 ' ' 1

Correlation of Fixed Effects:
            (Intr) h__r>0
hg___Dr>0.5 -0.353
round       -0.701 -0.111
optimizer (nloptwrap) convergence code: 0 (OK)
boundary (singular) fit: see help('isSingular')
```

Evaluating the effect of wealth visibility on inequality aversion punishment

```r
summary(glmer(punish_type_IA ~ showScore + round + (1|game) + (1|superid),
              data = exp1data %>% filter(round > 0),
              family = 'binomial', nAGQ=0,
      control = glmerControl(optimizer = c("bobyqa"),
                             optCtrl=list(maxfun=2e5),
                             calc.derivs=FALSE)))
```

```
Generalized linear mixed model fit by maximum likelihood (Adaptive
  Gauss-Hermite Quadrature, nAGQ = 0) [glmerMod]
 Family: binomial  ( logit )
Formula: punish_type_IA ~ showScore + round + (1 | game) + (1 | superid)
   Data: exp1data %>% filter(round > 0)
Control: glmerControl(optimizer = c("bobyqa"), optCtrl = list(maxfun = 2e+05),
    calc.derivs = FALSE)

     AIC      BIC   logLik deviance df.resid
  2115.4   2151.4  -1052.7   2105.4     9928

Scaled residuals:
    Min      1Q  Median      3Q     Max
-1.6785 -0.0963 -0.0821 -0.0726  4.9926

Random effects:
 Groups  Name        Variance Std.Dev.
 superid (Intercept) 4.6454   2.1553
 game    (Intercept) 0.6706   0.8189
Number of obs: 9933, groups:  superid, 719; game, 50

Fixed effects:
             Estimate Std. Error z value Pr(>|z|)
(Intercept) -4.626329   0.285225 -16.220   <2e-16 ***
showScore    0.233556   0.356549   0.655    0.512
round       -0.003266   0.015790  -0.207    0.836
---
Signif. codes:  0 '***' 0.001 '**' 0.01 '*' 0.05 '.' 0.1 ' ' 1

Correlation of Fixed Effects:
          (Intr) shwScr
showScore -0.656
round     -0.424  0.000
```

```r
summary(lmer(behaviorTime_sec ~ showScore + round + (1|game) + (1|superid),
             data = exp1data %>% filter(punish_type_IA == 1)))
```

```
boundary (singular) fit: see help('isSingular')
```

```
Linear mixed model fit by REML. t-tests use Satterthwaite's method [
lmerModLmerTest]
Formula: behaviorTime_sec ~ showScore + round + (1 | game) + (1 | superid)
   Data: exp1data %>% filter(punish_type_IA == 1)

REML criterion at convergence: 2074.9

Scaled residuals:
    Min      1Q  Median      3Q     Max
-2.0380 -0.4550 -0.2361  0.1051  5.8411

Random effects:
 Groups   Name        Variance Std.Dev.
 superid  (Intercept)  0.00    0.000
 game     (Intercept) 25.92    5.092
 Residual             71.95    8.482
Number of obs: 286, groups:  superid, 120; game, 43

Fixed effects:
             Estimate Std. Error        df t value Pr(>|t|)
(Intercept)   7.16246    1.78256  67.67375   4.018  0.00015 ***
showScore     1.09465    1.99383  35.39306   0.549  0.58644
round        -0.01564    0.13893 281.94491  -0.113  0.91043
---
Signif. codes:  0 '***' 0.001 '**' 0.01 '*' 0.05 '.' 0.1 ' ' 1

Correlation of Fixed Effects:
          (Intr) shwScr
showScore -0.573
round     -0.576 -0.043
optimizer (nloptwrap) convergence code: 0 (OK)
boundary (singular) fit: see help('isSingular')
```

Testing the reciprocal effect of defection for defection decisions

```
# Reciprocation occurs when previous round local rate is >0

exp1data = exp1data %>% mutate(any_defectors_lag =
                               ifelse(local_rate_defect_lag > 0, 1, 0))
exp2data = exp2data %>% mutate(any_defectors_lag =
                               ifelse(local_rate_defect_lag > 0, 1, 0))
```

```
summary(lmer(behaviorTime_sec ~ any_defectors_lag + round +
               (1|game) + (1|superid),
             data = exp1data %>% filter(behavior_defect == 1, round > 0)))
```

```
Linear mixed model fit by REML. t-tests use Satterthwaite's method [
lmerModLmerTest]
Formula: behaviorTime_sec ~ any_defectors_lag + round + (1 | game) + (1 |
    superid)
   Data: exp1data %>% filter(behavior_defect == 1, round > 0)

REML criterion at convergence: 28985.1

Scaled residuals:
    Min      1Q  Median      3Q     Max
-2.9714 -0.3557 -0.1635  0.0010  9.8659

Random effects:
 Groups   Name        Variance Std.Dev.
 superid  (Intercept) 15.0762  3.8828
 game     (Intercept)  0.8647  0.9299
 Residual             70.6379  8.4046
Number of obs: 4017, groups:  superid, 532; game, 50

Fixed effects:
                    Estimate Std. Error         df t value Pr(>|t|)
(Intercept)          7.37080    0.61965 1108.55638  11.895  < 2e-16 ***
any_defectors_lag   -0.44426    0.53732 3371.01222  -0.827 0.408406
round               -0.12143    0.03459 3880.05350  -3.511 0.000452 ***
---
Signif. codes:  0 '***' 0.001 '**' 0.01 '*' 0.05 '.' 0.1 ' ' 1

Correlation of Fixed Effects:
```

```
             (Intr) any_d_
any_dfctrs_ -0.771
round       -0.467  0.003
```

```r
summary(lmer(behaviorTime_sec ~ any_defectors_lag + round + (1|game) +
               (1|superid),
             data = exp2data %>% filter(behavior_defect == 1, round > 0,
                                        time_pressure == 'Minus')))
```

```
boundary (singular) fit: see help('isSingular')

Linear mixed model fit by REML. t-tests use Satterthwaite's method [
lmerModLmerTest]
Formula: behaviorTime_sec ~ any_defectors_lag + round + (1 | game) + (1 |
    superid)
   Data: exp2data %>% filter(behavior_defect == 1, round > 0, time_pressure ==
    "Minus")

REML criterion at convergence: 11007.6

Scaled residuals:
    Min      1Q  Median      3Q     Max
-3.2771 -0.3082 -0.1322  0.0909 16.1598

Random effects:
 Groups   Name        Variance Std.Dev.
 superid  (Intercept) 1.460    1.208
 game     (Intercept) 0.000    0.000
 Residual             2.951    1.718
Number of obs: 2693, groups:  superid, 259; game, 25

Fixed effects:
                   Estimate Std. Error         df t value Pr(>|t|)
(Intercept)        3.082e+00  1.933e-01  1.798e+03  15.947   <2e-16 ***
any_defectors_lag -5.897e-03  1.662e-01  2.671e+03  -0.035   0.9717
round             -1.464e-02  8.403e-03  2.472e+03  -1.742   0.0816 .
---
Signif. codes:  0 '***' 0.001 '**' 0.01 '*' 0.05 '.' 0.1 ' ' 1

Correlation of Fixed Effects:
            (Intr) any_d_
any_dfctrs_ -0.820
```

```
round       -0.408  0.050
optimizer (nloptwrap) convergence code: 0 (OK)
boundary (singular) fit: see help('isSingular')
```

```r
summary(lmer(behaviorTime_sec ~ any_defectors_lag + round + (1|game) +
               (1|superid),
             data = exp2data %>% filter(behavior_defect == 1, round > 0,
                                        time_pressure == 'Plus')))
```

```
Linear mixed model fit by REML. t-tests use Satterthwaite's method [
lmerModLmerTest]
Formula: behaviorTime_sec ~ any_defectors_lag + round + (1 | game) + (1 |
    superid)
   Data: exp2data %>% filter(behavior_defect == 1, round > 0, time_pressure ==
    "Plus")

REML criterion at convergence: 1190.8

Scaled residuals:
    Min      1Q  Median      3Q     Max
-2.4325 -0.6629 -0.0777  0.5550  3.7395

Random effects:
 Groups   Name        Variance Std.Dev.
 superid  (Intercept) 0.07516  0.2742
 game     (Intercept) 0.01126  0.1061
 Residual             0.07867  0.2805
Number of obs: 2174, groups:  superid, 247; game, 25

Fixed effects:
                    Estimate Std. Error         df t value Pr(>|t|)
(Intercept)        2.040e+00  4.621e-02  1.518e+02  44.135  < 2e-16 ***
any_defectors_lag -5.518e-04  3.486e-02  2.146e+03  -0.016    0.987
round             -7.010e-03  1.541e-03  1.972e+03  -4.550 5.69e-06 ***
---
Signif. codes:  0 '***' 0.001 '**' 0.01 '*' 0.05 '.' 0.1 ' ' 1

Correlation of Fixed Effects:
            (Intr) any_d_
any_dfctrs_ -0.722
round       -0.329  0.057
```

Evaluating the effect of choosing punishment on rewiring

```
## Regression for punishment and rewiring, Exp. 1 -------------------------------
model_e1_rwr = glmer(rewired ~ behavior_punish + round + (1|game) + (1|superid),
                     data = exp1data %>% filter(round > 0,
                                                behavior %in% c("C", "D", "P")),
                     family = 'binomial', nAGQ=0,
                     control = glmerControl(optimizer = c("bobyqa"),
                                            optCtrl=list(maxfun=2e5),
                                            calc.derivs=FALSE))
tidy(model_e1_rwr, exponentiate = T) %>% as.data.frame()
```

```
    effect   group            term  estimate   std.error   statistic
1    fixed    <NA>     (Intercept) 5.2864841 0.578111067  15.2268437
2    fixed    <NA> behavior_punish 0.9535831 0.114125479  -0.3971294
3    fixed    <NA>           round 0.9001853 0.005162444 -18.3360179
4 ran_pars superid sd__(Intercept) 0.8038238          NA          NA
5 ran_pars    game sd__(Intercept) 0.6347083          NA          NA
       p.value
1 2.346492e-52
2 6.912721e-01
3 4.269490e-75
4           NA
5           NA
```

```
## Regression for punishment and rewiring, Exp. 2 (TP-) ------------------------
model_e2_rwr_minus = glmer(rewired ~ behavior_punish + round + (1|game) +
                             (1|superid),
              data = exp2data %>% filter(round > 0,
                                         behavior %in% c('C', 'D', 'P'),
                                         time_pressure == 'Minus'),
              family = 'binomial', nAGQ=0,
              control = glmerControl(optimizer = c("bobyqa"),
                                     optCtrl=list(maxfun=2e5),
                                     calc.derivs=FALSE))
tidy(model_e2_rwr_minus, exponentiate = T) %>% as.data.frame()
```

```
    effect   group            term  estimate   std.error statistic      p.value
1    fixed    <NA>     (Intercept) 8.1130162 1.085044141 15.653145 3.161973e-55
2    fixed    <NA> behavior_punish 1.3454189 0.265622835  1.502857 1.328759e-01
```

```
3    fixed    <NA>           round 0.9448068 0.008581793 -6.250589 4.089089e-10
4 ran_pars superid sd__(Intercept) 1.4596213          NA        NA           NA
5 ran_pars    game sd__(Intercept) 0.3159409          NA        NA           NA
```

```
## Regression for punishment and rewiring, Exp. 2 (TP+) ------------------------
model_e2_rwr_plus = glmer(rewired ~ behavior_punish + round + (1|game) +
                            (1|superid),
              data = exp2data %>% filter(round > 0,
                                              behavior %in% c('C', 'D', 'P'),
                                              time_pressure == 'Plus'),
              family = 'binomial', nAGQ=0,
              control = glmerControl(optimizer = c("bobyqa"),
                                      optCtrl=list(maxfun=2e5),
                                      calc.derivs=FALSE))
tidy(model_e2_rwr_plus, exponentiate = T) %>% as.data.frame()
```

```
    effect   group            term  estimate   std.error  statistic
1    fixed    <NA>     (Intercept) 9.2522425 1.739726644 11.8323182
2    fixed    <NA> behavior_punish 1.1247841 0.248119669  0.5330676
3    fixed    <NA>           round 0.9408296 0.008932311 -6.4243497
4 ran_pars superid sd__(Intercept) 1.6406728          NA         NA
5 ran_pars    game sd__(Intercept) 0.6893328          NA         NA
       p.value
1 2.657009e-32
2 5.939868e-01
3 1.324345e-10
4           NA
5           NA
```

4. Figures

Figure 1 - Example of Player's Screen

```
example_fig1 = image_read('~/Documents/Projects/harming_esn/figures/fig1A.png') %>%
  image_ggplot() +
  labs(tag = 'A')
example_fig2 = image_read('~/Documents/Projects/harming_esn/figures/fig1B.png') %>%
  image_ggplot() +
  labs(tag = 'B')
```

```r
example_fig1 / example_fig2
```

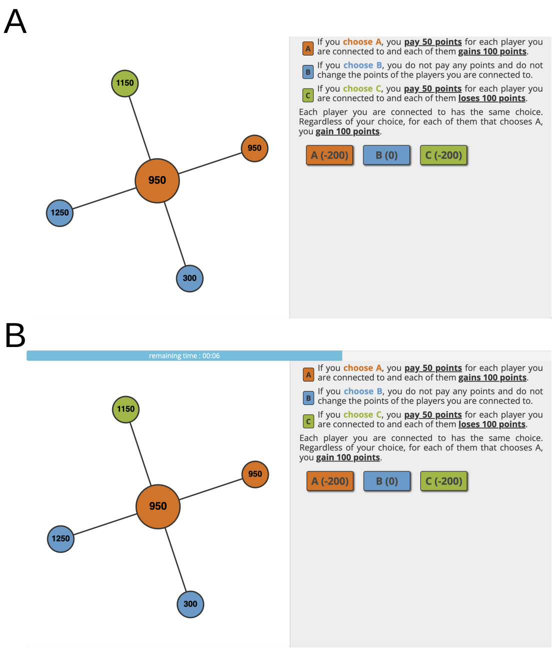


```r
# ggsave(filename = "~/Documents/Projects/harming_esn/figures/fig1combined.png",
#        width = 8, height = 8, units = "in")
```

Figure 2 - Behavior Distribution, Decision Times, and Punishment Mechanisms, Experiment 1

```r
# Fig 1A
exp1_fig1_data = bind_cols(data1_behavior_count, data1_behavior_CI, data1_times)[-7]

names(exp1_fig1_data) = c("behavior", "count", "crude_prop", "adjusted_prop",
                          "LL_prop", "UL_prop", "mean_dt", "se_mean_dt",
                          "UL_mean_dt", "LL_mean_dt")
exp1_fig1_data = exp1_fig1_data %>%
  mutate(behavior = case_match(behavior,
                               "C" ~ "Cooperation",
                               "D" ~ "Defection",
                               "P" ~ "Punishment"))
```

```
exp1_fig1_A = exp1_fig1_data %>%
  ggplot() +
  aes(x = behavior, y = adjusted_prop, fill = behavior) +
  geom_bar(stat = "identity", width = 0.75, alpha = 0.5) +
  geom_errorbar(aes(ymin = LL_prop, ymax = UL_prop, color = factor(behavior)),
                width = 0.25) +
  theme_classic() +
  scale_y_continuous(limits = c(0, 0.6), breaks = seq(0, 0.6, by = 0.2),
                     expand = c(0, 0)) +
  ylab("Proportion") +
  scale_fill_manual(values = c("#00A5CF", "#FFBF00", "#574AE2")) +
  scale_color_manual(values = c("dodgerblue4", "darkorange3", "#574AE2")) +
  labs(tag = "A") +
  theme(panel.grid.minor = element_blank(),
        panel.grid.major = element_blank(),
        legend.position = "none",
        axis.text.x = element_text(size = 9),
        axis.title.x = element_blank(),
        axis.ticks.x = element_blank(),
        axis.ticks.y = element_blank())
exp1_fig1_A
```

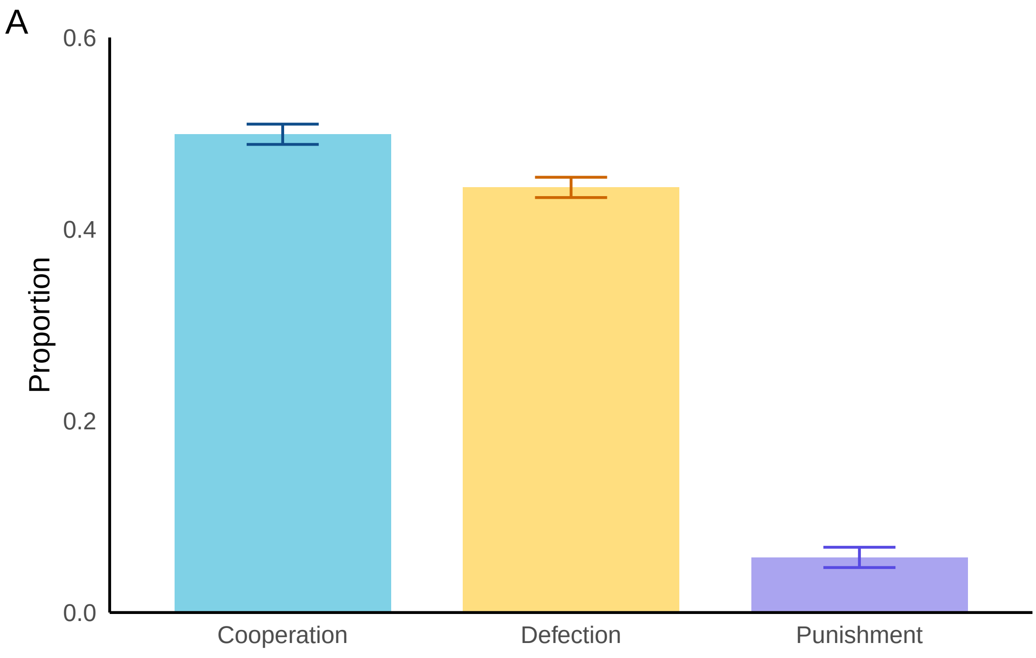

```
exp1_fig1_B = exp1_fig1_data %>%
  ggplot() +
  aes(x = behavior, y = mean_dt, fill = behavior) +
  geom_bar(stat = "identity", width = 0.75, alpha = 0.5) +
  geom_errorbar(aes(ymin = mean_dt - 1.96*se_mean_dt,
                    ymax = mean_dt + 1.96*se_mean_dt,
                    color = factor(behavior)), width = 0.25) +
  theme_classic() +
  scale_y_continuous(limits = c(0, 8.5), breaks = seq(0, 8, by = 2),
                     expand = c(0, 0)) +
  ylab("Decision time \n (seconds)")+
  scale_fill_manual(values = c("#00A5CF", "#FFBF00", "#574AE2")) +
  scale_color_manual(values = c("dodgerblue4", "darkorange3", "#574AE2")) +
  labs(tag = "B") +
  theme(panel.grid.minor = element_blank(),
        panel.grid.major = element_blank(),
        legend.position = "none",
        axis.text.x = element_text(size = 9),
        axis.title.x = element_blank(),
        axis.ticks.x = element_blank(),
        axis.ticks.y = element_blank())
exp1_fig1_B
```

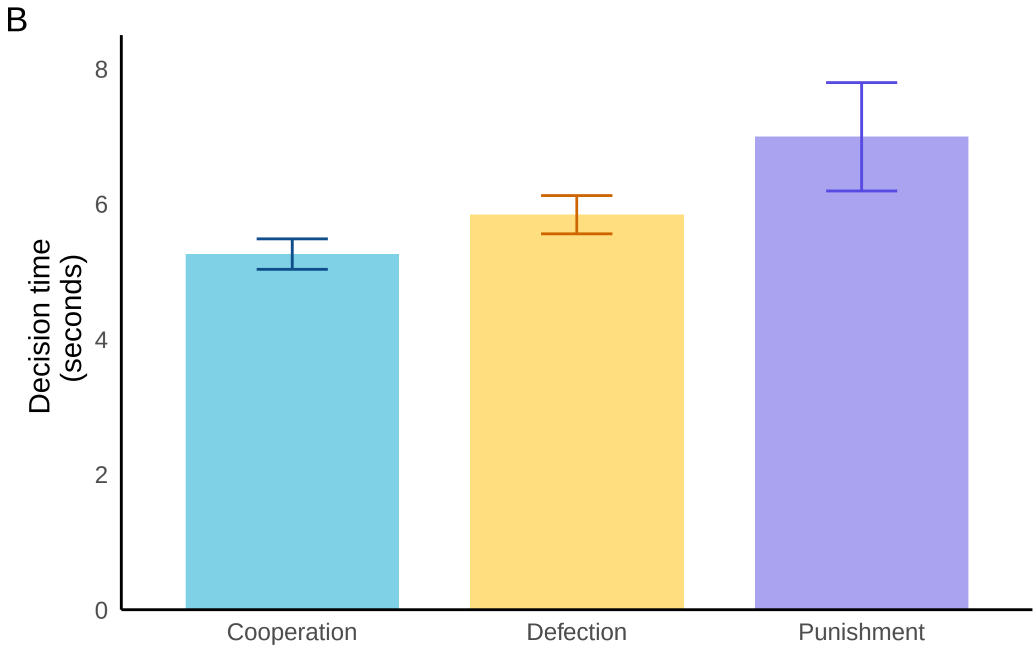

```
exp1data_NR = exp1data %>%
  group_by(punish_type_NR) %>%
  count() %>%
  ungroup() %>%
  mutate(punish_type = "NR",
         total = sum(n),
         perc = n/sum(n),
         se_perc = sqrt((perc*(1-perc))/total)) %>%
  filter(punish_type_NR == 1)  %>%
  select(punish_type, n, total, perc, se_perc)

exp1data_IA = exp1data %>%
  group_by(punish_type_IA) %>%
  count() %>%
  ungroup() %>%
  mutate(punish_type = "IA",
         total = sum(n),
         perc = n/sum(n),
         se_perc = sqrt((perc*(1-perc))/total)) %>%
  filter(punish_type_IA == 1)  %>%
  select(punish_type, n, total, perc, se_perc)

exp1data_CR = exp1data %>%
  group_by(punish_type_CR) %>%
  count() %>%
  ungroup() %>%
  mutate(punish_type = "CR",
         total = sum(n),
         perc = n/sum(n),
         se_perc = sqrt((perc*(1-perc))/total)) %>%
  filter(punish_type_CR == 1)  %>%
  select(punish_type, n, total, perc, se_perc)

exp1data_U = exp1data %>%
  group_by(punish_type_U) %>%
  count() %>%
  ungroup() %>%
  mutate(punish_type = "U",
         total = sum(n),
         perc = n/sum(n),
         se_perc = sqrt((perc*(1-perc))/total)) %>%
  filter(punish_type_U == 1)  %>%
```

```
  select(punish_type, n, total, perc, se_perc)

figS1data = bind_rows(exp1data_CR, exp1data_IA, exp1data_NR, exp1data_U)

figS1data = figS1data %>%
  mutate(perc_LL = perc - 1.96*se_perc,
         perc_UL = perc + 1.96*se_perc)
```

```
# Decision times by punish type

exp1data_NR_times = exp1data %>%
  filter(punish_type_NR == 1, behavior_punish == 1) %>%
  summarize(mean_dt = mean1(behaviorTime_sec),
            se_mean_dt = se_mean(behaviorTime_sec)) %>%
  mutate(punish_type = "NR",
         mean_LL = mean_dt - 1.96*se_mean_dt,
         mean_UL = mean_dt + 1.96*se_mean_dt)

exp1data_IA_times = exp1data %>%
  filter(punish_type_IA == 1, behavior_punish == 1) %>%
  summarize(mean_dt = mean1(behaviorTime_sec),
            se_mean_dt = se_mean(behaviorTime_sec),
) %>%
  mutate(punish_type = "IA",   mean_LL = mean_dt - 1.96*se_mean_dt,
         mean_UL = mean_dt + 1.96*se_mean_dt)

exp1data_CR_times = exp1data %>%
  filter(punish_type_CR == 1, behavior_punish == 1) %>%
  summarize(mean_dt = mean1(behaviorTime_sec),
            se_mean_dt = se_mean(behaviorTime_sec)) %>%
  mutate(punish_type = "CR",
           mean_LL = mean_dt - 1.96*se_mean_dt,
         mean_UL = mean_dt + 1.96*se_mean_dt)

exp1data_U_times = exp1data %>%
  filter(punish_type_U == 1, behavior_punish == 1) %>%
  summarize(mean_dt = mean1(behaviorTime_sec),
            se_mean_dt = se_mean(behaviorTime_sec)) %>%
  mutate(punish_type = "U",
         mean_LL = mean_dt - 1.96*se_mean_dt,
         mean_UL = mean_dt + 1.96*se_mean_dt)
```

```
exp1data_punish_types_times = bind_rows(exp1data_NR_times,
                                        exp1data_CR_times,
                                        exp1data_IA_times,
                                        exp1data_U_times) %>%
  select(punish_type, mean_dt, se_mean_dt, mean_LL, mean_UL)

fig1data = figS1data %>%
  left_join(exp1data_punish_types_times, by = "punish_type") %>%
  mutate(punish_type = case_match(punish_type,
              "CR" ~ "Copying/retaliation",
              "IA" ~ "Inequality aversion",
              "NR" ~ "Negative reinforcement",
              "U" ~ "Unclassified")) %>%
  mutate(punish_type_fct = factor(punish_type, levels = c("Copying/retaliation", "Negative re
```

```
exp1_fig1_C = fig1data %>%
  ggplot(aes(x = punish_type_fct, y = perc, fill = punish_type_fct)) +
  geom_bar(position = "dodge", stat = "identity", alpha = 0.5, show.legend = F) +
  geom_errorbar(aes(ymin = perc + 1.96*se_perc,
                    ymax = perc - 1.96*se_perc,
                    width = 0.25),
                color = "purple4",
                position = position_dodge(.9),
                show.legend = F) +
  scale_fill_manual(values = c("Copying/retaliation" = "plum3",
                               "Negative reinforcement" = "mediumslateblue",
                               "Inequality aversion" = "mediumpurple3",
                               "Unclassified" = "orchid4"), guide = "none") +
  scale_color_manual(guide = "none") +
  scale_x_discrete(labels = c("Copying/\nretaliation", "Negative\nreinforcement",
                              "Inequality\naversion", "Unclassified")) +
  scale_y_continuous(limits = c(0, 0.05), expand = c(0, 0)) +
  ylab("Proportion") +
  xlab("") +
  labs(tag = "C") +
  theme_classic() +
  theme(panel.grid.minor = element_blank(),
        panel.grid.major = element_blank(),
        legend.position = "bottom",
        legend.title = element_blank(),
        axis.text.x = element_text(size = 8),
        axis.title.x = element_blank(),
```

```
        axis.ticks.x = element_blank(),
        axis.ticks.y = element_blank())
exp1_fig1_C
```

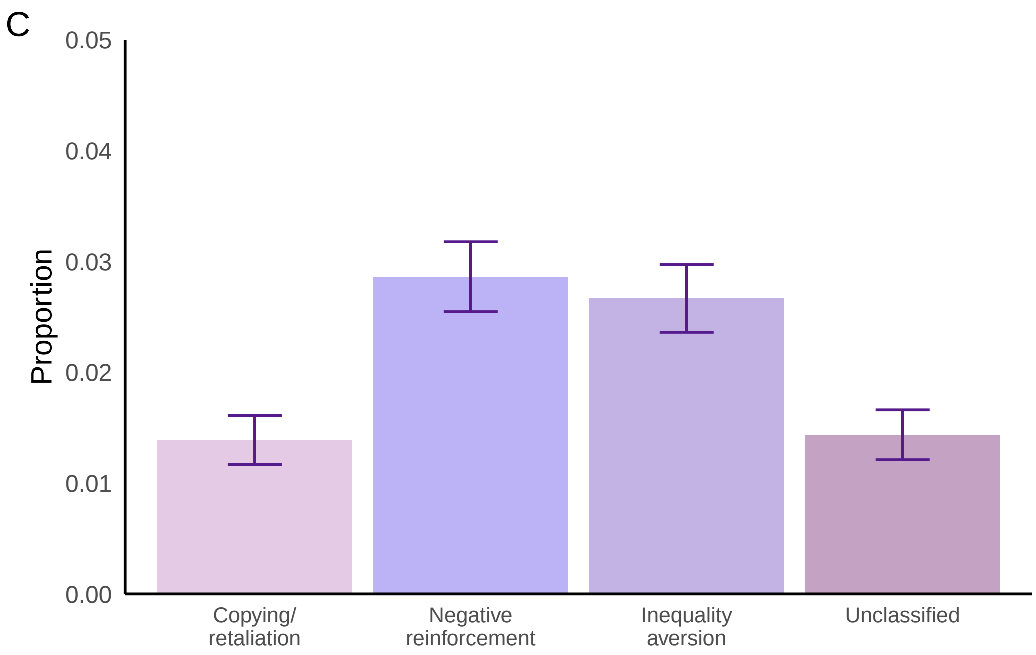


```
exp1_fig1_D = fig1data %>%
  ggplot(aes(x = punish_type_fct, y = mean_dt, fill = punish_type_fct)) +
  geom_bar(stat = "identity", width = 0.75,  alpha = 0.5) +
  geom_errorbar(aes(ymin = mean_LL,
                    ymax = mean_UL),
                color = "purple4", width = 0.25) +
  theme_classic() +
  scale_y_continuous(limits = c(0, 10), breaks = seq(0, 10, by = 2), expand = c(0, 0)) +
  ylab("Decision time \n (seconds)")+
  scale_fill_manual(values = c("Copying/retaliation" = "plum3",
                               "Negative reinforcement" = "mediumslateblue",
                               "Inequality aversion" = "mediumpurple3",
                               "Unclassified" = "orchid4"), guide = "none") +
  scale_x_discrete(labels = c("Copying/\nretaliation", "Negative\nreinforcement",
                              "Inequality\naversion", "Unclassified")) +
  scale_color_manual(guide = "none") +
  theme_classic() +
  labs(tag = "D") +
```

```
  theme(panel.grid.minor = element_blank(),
        panel.grid.major = element_blank(),
        legend.position = "none",
        axis.text.x = element_text(size = 8),
        axis.title.x = element_blank(),
        axis.ticks.x = element_blank(),
        axis.ticks.y = element_blank())
exp1_fig1_D
```

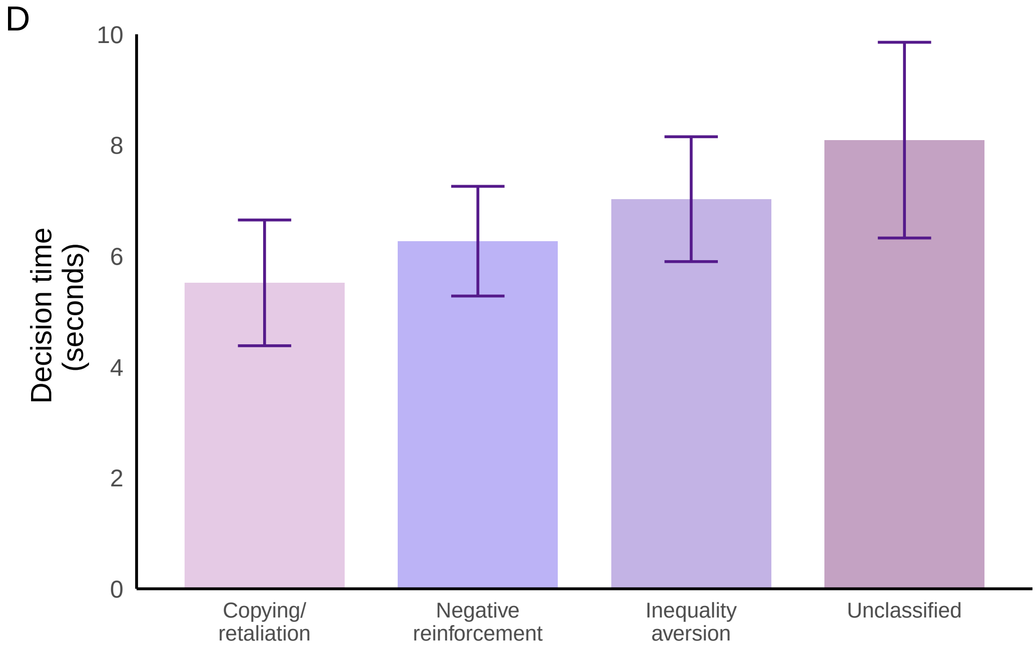

Main

```
(exp1_fig1_A + exp1_fig1_B)/ (exp1_fig1_C + exp1_fig1_D)
```

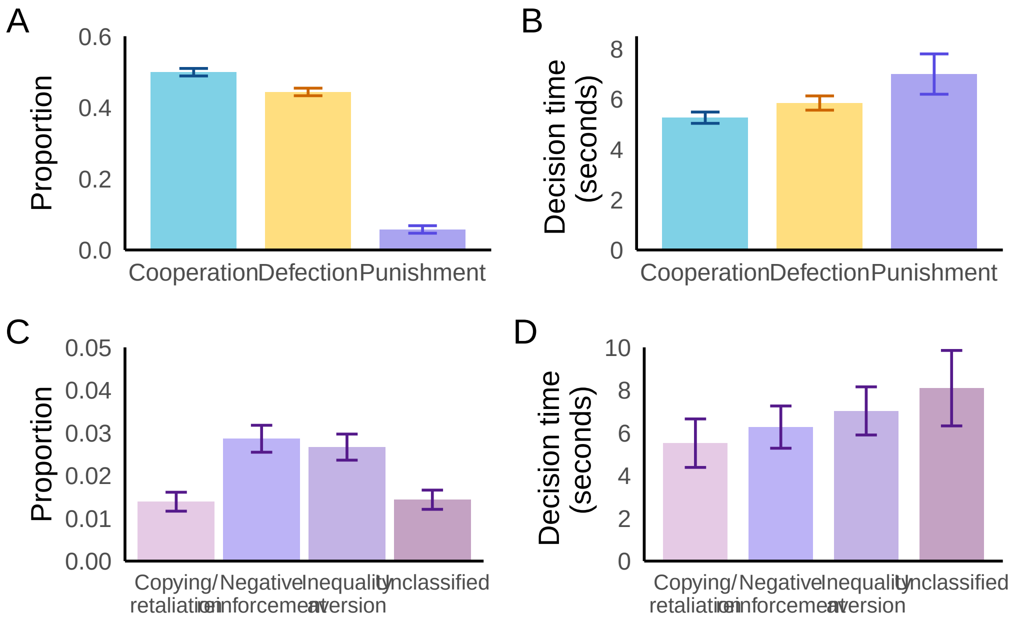


```
# ggsave(filename = "~/Documents/Projects/harming_esn/figures/fig2.png",
#        width = 8, height = 8, units = "in")
```

Figure 3 - Behavior Distribution, Decision Times, and Punishment Mechanisms, Experiment 2

```
exp2_tp_plus_fig3_data = bind_cols(exp2data_tp_plus_count, exp2data_tp_plus_CI,
                                    exp2data_tp_plus_times)[-7]
names(exp2_tp_plus_fig3_data) = c("behavior", "count", "crude_prop",
                                   "adjusted_prop", "LL_prop", "UL_prop", "mean_dt",
                                   "se_mean_dt", "LL_mean_dt", "UL_mean_dt")
exp2_tp_plus_fig3_data$setting = "TP+"

exp2_tp_minus_fig3_data = bind_cols(exp2data_tp_minus_count, exp2data_tp_minus_CI,
                                     exp2data_tp_minus_times)[-7]
names(exp2_tp_minus_fig3_data) = c("behavior", "count", "crude_prop",
                                   "adjusted_prop", "LL_prop", "UL_prop", "mean_dt",
                                   "se_mean_dt", "LL_mean_dt", "UL_mean_dt")

exp2_tp_minus_fig3_data$setting = "TP-"
```

```
fig3data = bind_rows(exp2_tp_minus_fig3_data, exp2_tp_plus_fig3_data)

fig3data = fig3data %>%
  mutate(behavior = case_match(behavior, "C" ~ "Cooperation",
                               "D" ~ "Defection", "P" ~ "Punishment"))
```

Main

```
fig3A = fig3data %>%
  ggplot(aes(x = behavior, y = adjusted_prop, fill = behavior, pattern = setting)) +
  geom_bar_pattern(position = "dodge", stat = "identity", alpha = 0.5,
                   pattern_density = 0.4, pattern_color = "white",
                   pattern_shape = 3) +
  geom_errorbar(aes(ymin = LL_prop,
                    ymax = UL_prop,
                    color = behavior,
                    width = 0.25),
                position = position_dodge(.9),
                show.legend = F) +
  scale_pattern_manual(values = c("none", "pch")) +
  scale_fill_manual(values = c("Cooperation" = "#00A5CF", "Defection" = "#FFBF00",
                               "Punishment" = "#574AE2")) +
  scale_color_manual(values = c("dodgerblue4", "darkorange3", "#574AE2")) +
  scale_y_continuous(limits = c(0, 0.65), expand = c(0, 0)) +
  guides(color = "none", fill = "none", pattern = guide_legend(title = "Setting")) +
  ylab("Proportion") +
  xlab("") +
  theme_classic() +
  labs(tag = "A") +
  theme(panel.grid.minor = element_blank(),
        panel.grid.major = element_blank(),
        axis.text.x = element_text(size = 10),
        axis.title.x = element_blank(),
        axis.ticks.x = element_blank(),
        axis.ticks.y = element_blank(),
        legend.position = "bottom",
        legend.key.size = unit(1, "cm"),
        legend.key = element_rect(fill = "#574AE2", color = NA)) +
  annotate("text", x = 1:3,
           y = c(0.46, 0.61, 0.12),
```

```
          label = c("italic(p) == 0.661", "italic(p) == 0.758", "italic(p) == 0.475"),
          parse = T)
fig3A
```

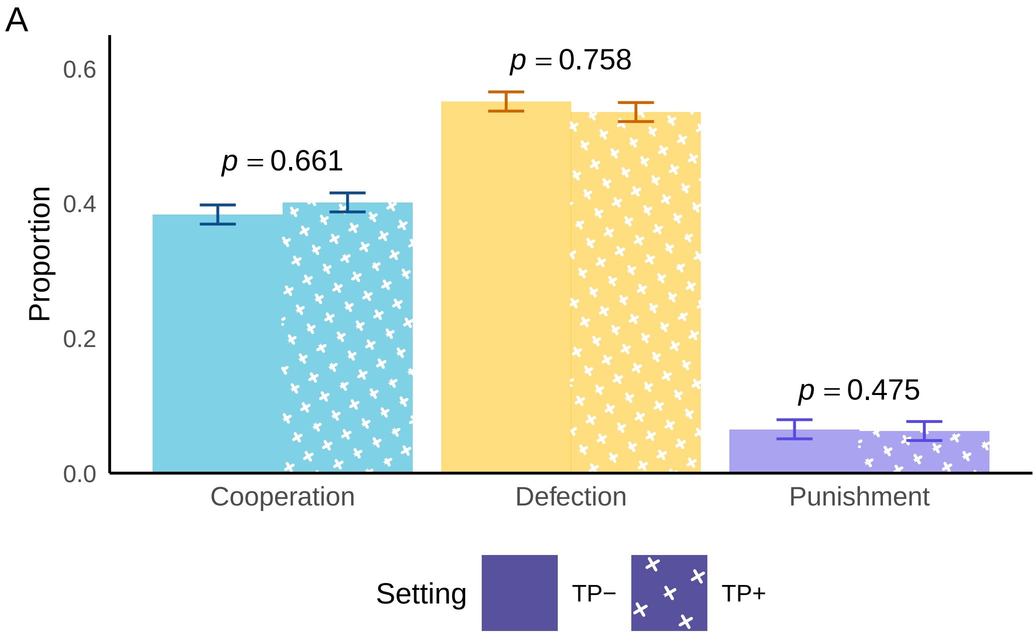


```
fig3B = fig3data %>%
  ggplot() +
  aes(x = behavior, y = mean_dt, fill = behavior, pattern = setting) +
  geom_bar_pattern(stat = "identity", width = 0.75, alpha = 0.5,
                   position = "dodge",
                   pattern_density = 0.4,
                   pattern_color = "white",
                   pattern_shape = 3, show.legend = F) +
  geom_errorbar(aes(ymin = mean_dt - se_mean_dt,
                    ymax = mean_dt + se_mean_dt,
                    color = factor(behavior)),
                    position = position_dodge(0.75),
                    width = 0.25,
                show.legend = F) +
  geom_hline(yintercept = 3, color = "red2", alpha = 0.3) +
  theme_classic() +
  scale_pattern_manual(values = c("none", "pch")) +
  scale_y_continuous(limits = c(0, 5), expand = c(0, 0)) +
```

```
  scale_x_discrete(limits = c("Cooperation", "Defection", "Punishment")) +
  ylab("Decision time \n (seconds)")+
  scale_fill_manual(values = c("#00A5CF", "#FFBF00", "#574AE2")) +
  scale_color_manual(values = c("dodgerblue4", "darkorange3", "#574AE2")) +
  labs(tag = "B") +
  theme(panel.grid.minor = element_blank(),
        panel.grid.major = element_blank(),
        legend.position = "none",
        axis.text.x = element_text(size = 10),
        axis.title.x = element_blank(),
        axis.ticks.x = element_blank(),
        axis.ticks.y = element_blank()) +
    annotate("text", x = 1:3,
           y = c(3.45, 3.25, 4.25),
           label = c(rep(expression(paste(italic("p")," < 0.0001")), 3)))

fig3B
```

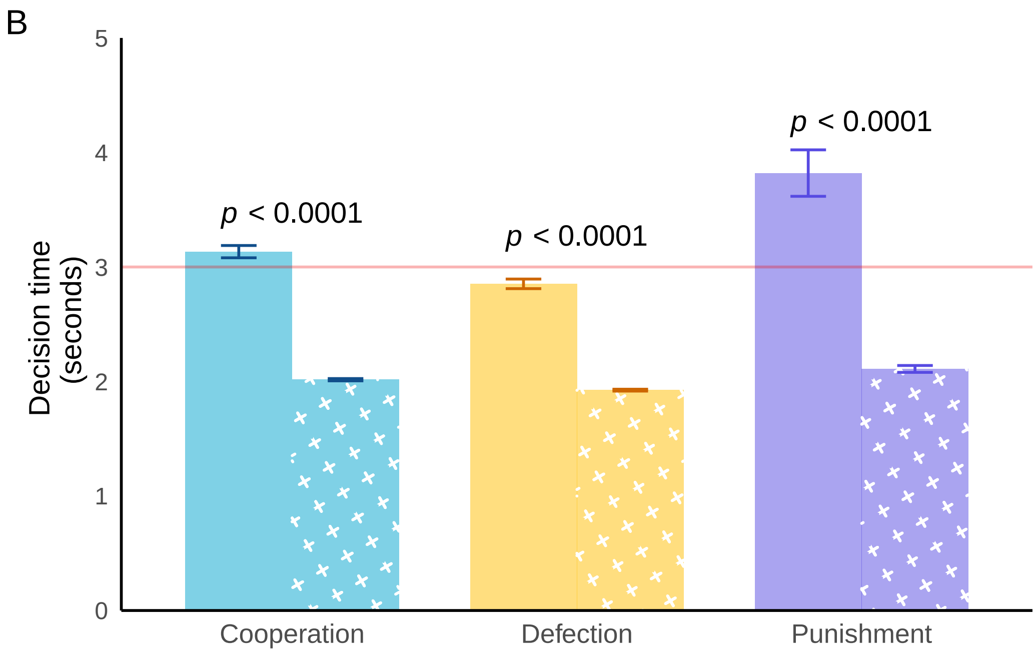


```
fig3A + fig3B +
  plot_annotation(tag_levels = c("A", "B")) +
  plot_layout(guides = "collect") &
  theme(legend.position = "bottom")
```

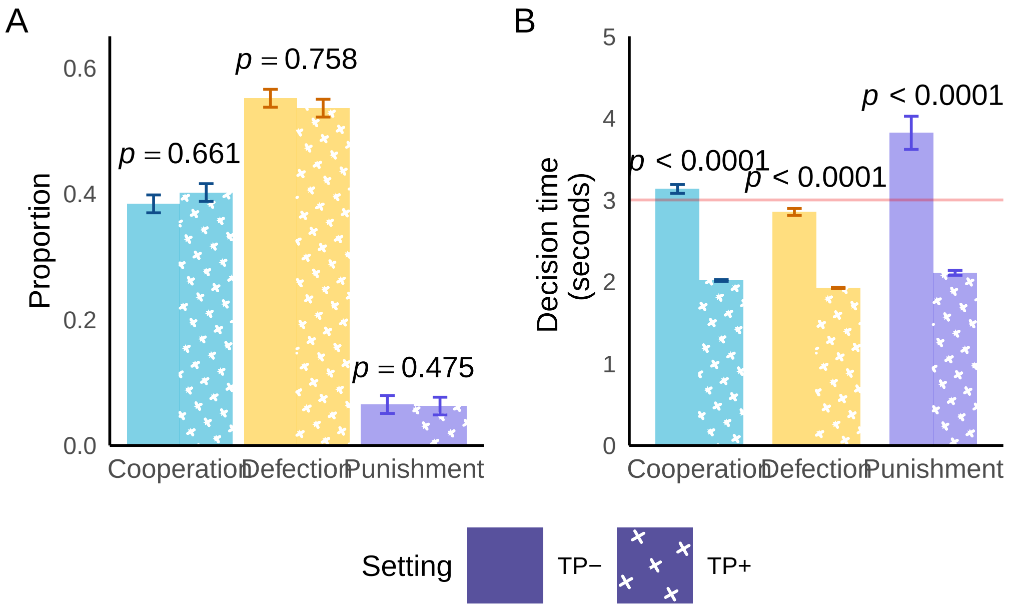


```
#ggsave(filename = "~/Documents/Projects/harming_esn/figures/fig3.png",
#width =  7, height = 5, units = "in")
```

Figure 4 - Punishment Mechanism Decision Times, Experiment 2

```
# Get the frequencies stratified by TP status and by punishment type
exp2data = exp2data %>%
  mutate(punish_type_U =
           ifelse(is.na(punish_type_CR) == 1 & is.na(punish_type_NR) == 1 |
                    is.na(punish_type_IA) == 1 & is.na(punish_type_U) == 1,
                  1, punish_type_U),
         punish_type_CR = ifelse(punish_type_U == 1, 0, punish_type_CR),
         punish_type_NR = ifelse(punish_type_U == 1, 0, punish_type_NR),
         punish_type_IA = ifelse(punish_type_U == 1, 0, punish_type_IA))

fig4_tp_minus_CR = exp2data %>%
  filter(time_pressure == "Minus") %>%
  group_by(punish_type_CR) %>%
  count() %>%
  ungroup() %>%
  mutate(setting = "TP-",
```

```
          punish_type = "CR",
          total = sum(n),
          perc = n/sum(n),
          se_perc = sqrt((perc*(1-perc))/total)) %>%
  filter(punish_type_CR == 1)  %>%
  select(setting, punish_type, n, total, perc, se_perc)

fig4_tp_minus_IA = exp2data %>%
  filter(time_pressure == "Minus") %>%
  group_by(punish_type_IA) %>%
  count() %>%
  ungroup() %>%
  mutate(setting = "TP-",
          punish_type = "IA",
          total = sum(n),
          perc = n/sum(n),
          se_perc = sqrt((perc*(1-perc))/total)) %>%
  filter(punish_type_IA == 1)  %>%
  select(setting, punish_type, n, total, perc, se_perc)

fig4_tp_minus_NR = exp2data %>%
  filter(time_pressure == "Minus") %>%
  group_by(punish_type_NR) %>%
  count() %>%
  ungroup() %>%
  mutate(setting = "TP-",
          punish_type = "NR",
          total = sum(n),
          perc = n/sum(n),
          se_perc = sqrt((perc*(1-perc))/total)) %>%
  filter(punish_type_NR == 1)  %>%
  select(setting, punish_type, n, total, perc, se_perc)

fig4_tp_minus_U = exp2data %>%
  filter(time_pressure == "Minus") %>%
  group_by(punish_type_U) %>%
  count() %>%
  ungroup() %>%
  mutate(setting = "TP-",
          punish_type = "U",
          total = sum(n),
          perc = n/sum(n),
```

```
        se_perc = sqrt((perc*(1-perc))/total)) %>%
  filter(punish_type_U == 1)  %>%
  select(setting, punish_type, n, total, perc, se_perc)

fig4_tp_minus_data = bind_rows(fig4_tp_minus_CR, fig4_tp_minus_IA,
                               fig4_tp_minus_NR, fig4_tp_minus_U)
```

```
fig4_tp_Plus_CR = exp2data %>%
  filter(time_pressure == "Plus") %>%
  group_by(punish_type_CR) %>%
  count() %>%
  ungroup() %>%
  mutate(setting = "TP+",
         punish_type = "CR",
         total = sum(n),
         perc = n/sum(n),
         se_perc = sqrt((perc*(1-perc))/total)) %>%
  filter(punish_type_CR == 1)  %>%
  select(setting, punish_type, n, total, perc, se_perc)

fig4_tp_Plus_IA = exp2data %>%
  filter(time_pressure == "Plus") %>%
  group_by(punish_type_IA) %>%
  count() %>%
  ungroup() %>%
  mutate(setting = "TP+",
         punish_type = "IA",
         total = sum(n),
         perc = n/sum(n),
         se_perc = sqrt((perc*(1-perc))/total)) %>%
  filter(punish_type_IA == 1)  %>%
  select(setting, punish_type, n, total, perc, se_perc)

fig4_tp_Plus_NR = exp2data %>%
  filter(time_pressure == "Plus") %>%
  group_by(punish_type_NR) %>%
  count() %>%
  ungroup() %>%
  mutate(setting = "TP+",
         punish_type = "NR",
         total = sum(n),
         perc = n/sum(n),
```

```
         se_perc = sqrt((perc*(1-perc))/total)) %>%
  filter(punish_type_NR == 1)  %>%
  select(setting, punish_type, n, total, perc, se_perc)

fig4_tp_Plus_U = exp2data %>%
  filter(time_pressure == "Plus") %>%
  group_by(punish_type_U) %>%
  count() %>%
  ungroup() %>%
  mutate(setting = "TP+",
         punish_type = "U",
         total = sum(n),
         perc = n/sum(n),
         se_perc = sqrt((perc*(1-perc))/total)) %>%
  filter(punish_type_U == 1)  %>%
  select(setting, punish_type, n, total, perc, se_perc)

fig4_tp_Plus_data = bind_rows(fig4_tp_Plus_CR, fig4_tp_Plus_IA,
                              fig4_tp_Plus_NR, fig4_tp_Plus_U)

exp2data_combined = bind_rows(fig4_tp_minus_data, fig4_tp_Plus_data)
```

```
exp2data_plus_NR_times = exp2data %>%
  filter(punish_type_NR == 1,
         behavior_punish == 1,
         time_pressure == "Plus") %>%
  summarize(mean_dt = mean1(behaviorTime_sec),
            se_mean_dt = se_mean(behaviorTime_sec)) %>%
  mutate(punish_type = "NR",
         setting = "TP+",
           mean_LL = mean_dt - 1.96*se_mean_dt,
         mean_UL = mean_dt + 1.96*se_mean_dt)

exp2data_plus_CR_times = exp2data %>%
  filter(punish_type_CR == 1,
         behavior_punish == 1,
         time_pressure == "Plus") %>%
  summarize(mean_dt = mean1(behaviorTime_sec),
            se_mean_dt = se_mean(behaviorTime_sec)) %>%
  mutate(punish_type = "CR",
         setting = "TP+",
         mean_LL = mean_dt - 1.96*se_mean_dt,
```

```
        mean_UL = mean_dt + 1.96*se_mean_dt)

exp2data_plus_IA_times = exp2data %>%
  filter(punish_type_IA == 1,
         behavior_punish == 1,
         time_pressure == "Plus") %>%
  summarize(mean_dt = mean1(behaviorTime_sec),
            se_mean_dt = se_mean(behaviorTime_sec)) %>%
  mutate(punish_type = "IA",
                  setting = "TP+",
           mean_LL = mean_dt - 1.96*se_mean_dt,
         mean_UL = mean_dt + 1.96*se_mean_dt)

exp2data_plus_U_times = exp2data %>%
  filter(punish_type_U == 1,
         behavior_punish == 1,
         time_pressure == "Plus") %>%
  summarize(mean_dt = mean1(behaviorTime_sec),
            se_mean_dt = se_mean(behaviorTime_sec)) %>%
  mutate(punish_type = "U",
         setting = "TP+",
         mean_LL = mean_dt - 1.96*se_mean_dt,
         mean_UL = mean_dt + 1.96*se_mean_dt)

exp2data_minus_NR_times = exp2data %>%
  filter(punish_type_NR == 1,
         behavior_punish == 1,
         time_pressure == "Minus") %>%
  summarize(mean_dt = mean1(behaviorTime_sec),
            se_mean_dt = se_mean(behaviorTime_sec)) %>%
  mutate(punish_type = "NR",
                  setting = "TP-",
           mean_LL = mean_dt - 1.96*se_mean_dt,
         mean_UL = mean_dt + 1.96*se_mean_dt)

exp2data_minus_CR_times = exp2data %>%
  filter(punish_type_CR == 1,
         behavior_punish == 1,
         time_pressure == "Minus") %>%
  summarize(mean_dt = mean1(behaviorTime_sec),
            se_mean_dt = se_mean(behaviorTime_sec)) %>%
  mutate(punish_type = "CR",
```

```
         setting = "TP-",
        mean_LL = mean_dt - 1.96*se_mean_dt,
         mean_UL = mean_dt + 1.96*se_mean_dt)

exp2data_minus_IA_times = exp2data %>%
  filter(punish_type_IA == 1,
         behavior_punish == 1,
         time_pressure == "Minus") %>%
  summarize(mean_dt = mean1(behaviorTime_sec),
            se_mean_dt = se_mean(behaviorTime_sec)) %>%
  mutate(punish_type = "IA",
         setting = "TP-",
         mean_LL = mean_dt - 1.96*se_mean_dt,
         mean_UL = mean_dt + 1.96*se_mean_dt)

exp2data_minus_U_times = exp2data %>%
  filter(punish_type_U == 1,
         behavior_punish == 1,
         time_pressure == "Minus") %>%
  summarize(mean_dt = mean1(behaviorTime_sec),
            se_mean_dt = se_mean(behaviorTime_sec)) %>%
  mutate(punish_type = "U",
         setting = "TP-",
         mean_LL = mean_dt - 1.96*se_mean_dt,
         mean_UL = mean_dt + 1.96*se_mean_dt)

exp2data_times = bind_rows(exp2data_minus_CR_times, exp2data_minus_IA_times,
                           exp2data_minus_NR_times, exp2data_minus_U_times,
                           exp2data_plus_CR_times, exp2data_plus_IA_times,
                           exp2data_plus_NR_times, exp2data_plus_U_times)
```

Main

```
# Create the combined figure
exp2data_combined = exp2data_combined %>%
         mutate(punish_type_fct = factor(punish_type,
                                         levels = c("CR", "NR", "IA", "U")))

exp2data_combined %>%
  ggplot(aes(x = punish_type_fct, y = perc, fill = punish_type_fct,
```

```r
           pattern = setting)) +
  geom_bar_pattern(position = "dodge", stat = "identity", alpha = 0.5,
                   pattern_density = 0.25, pattern_color = "white",
                   pattern_shape = 3) +
  geom_errorbar(aes(ymin = perc + 1.96*se_perc,
                    ymax = perc - 1.96*se_perc,
                    width = 0.25),
                    color = "purple4",
                position = position_dodge(.9),
                show.legend = F) +
  scale_pattern_manual(values = c("none", "pch")) +
  guides(color = "none", fill = "none",
         pattern = guide_legend(title = "Setting")) +
  annotate("text", x = 1:4,
           y = c(0.025, 0.0425, 0.045, 0.02),
           label = c("italic(p) == 0.889", "italic(p) == 0.629",
                     "italic(p) == 0.435", "italic(p) == 0.649"),
           parse = T) +
  scale_fill_manual(values = c("CR" = "plum3",
                               "NR" = "mediumslateblue",
                               "IA" = "mediumpurple3",
                               "U" = "orchid4"),
                    limits = c("CR", "NR", "IA", "U"),
                    guide = "none") +
  scale_x_discrete(labels = c("Copying/\nretaliation", "Negative\nreinforcement",
                              "Inequality\naversion", "Unclassified")) +
  scale_y_continuous(limits = c(0, 0.05), expand = c(0, 0)) +
  ylab("Proportion") +
  xlab("") +
  theme_classic() +
  theme(panel.grid.minor = element_blank(),
        panel.grid.major = element_blank(),
        legend.position = "bottom",
        legend.key.size = unit(1, "cm"),
        legend.key = element_rect(fill = "#574AE2", color = NA),
        axis.title.x = element_blank(),
        axis.ticks.x = element_blank(),
        axis.ticks.y = element_blank())
```

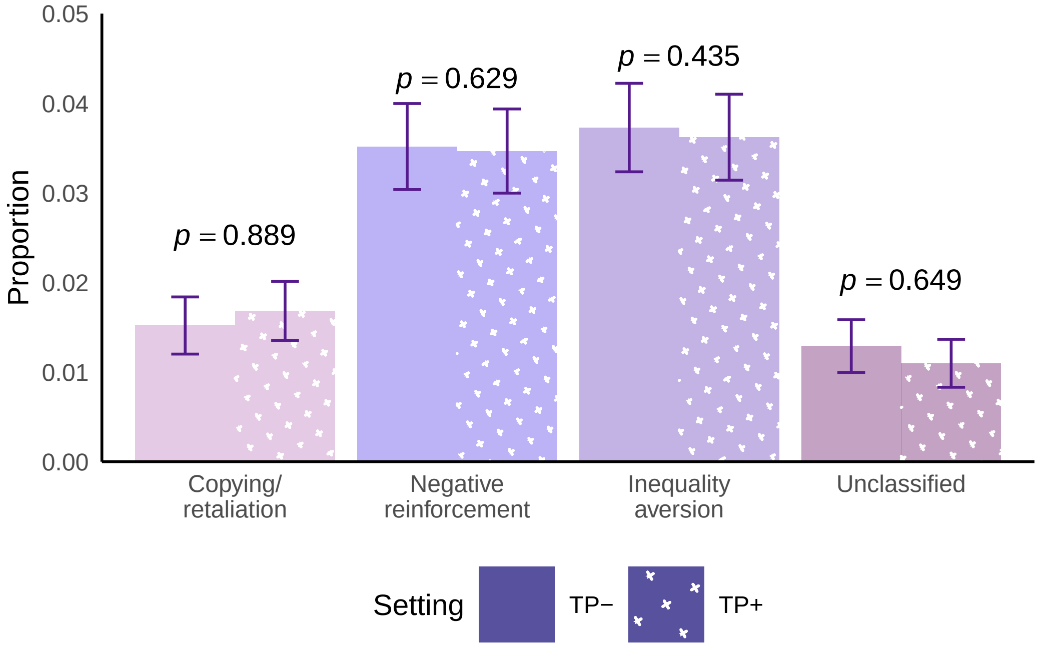


```
# ggsave(filename = "~/Documents/Projects/harming_esn/figures/fig4.png",
# width = 7, height = 5, units = "in")
```

Figure S2 - Distribution of Decision Times, Experiment 1

```
exp1data %>%
  filter(is.na(behaviorTime_prompt/1000) == 0, behavior %in% c("C", "D", "P")) %>%
  ggplot(aes(x = behaviorTime_prompt/1000, color = behavior)) +
  geom_density(adjust = 2, key_glyph = "path") +
  geom_vline(xintercept = 3, color = "red2", linetype = "dashed") +
  theme_classic() +
  labs(color = "Behavior") +
  xlab("Decision Time (sec)") +
  scale_x_log10(limits = c(1, 110),breaks = c(1, 10, 100),
              name = "Decision Time (sec)") +
  scale_y_continuous(limits = c(0, 2), name = "Density") +
  scale_color_manual(labels = c("Cooperation", "Defection", "Punishment"),
                   values = c("#00A5CF", "#FFBF00", "#574AE2"), guide = "none") +
  guides(colour=guide_legend(title = NULL)) +
  theme(panel.grid.minor = element_blank(),
        panel.grid.major = element_blank(),
```

```
        legend.position = c(0.9, 0.9),
        legend.title = element_blank(),
        axis.ticks.x = element_blank(),
        axis.ticks.y = element_blank())
```

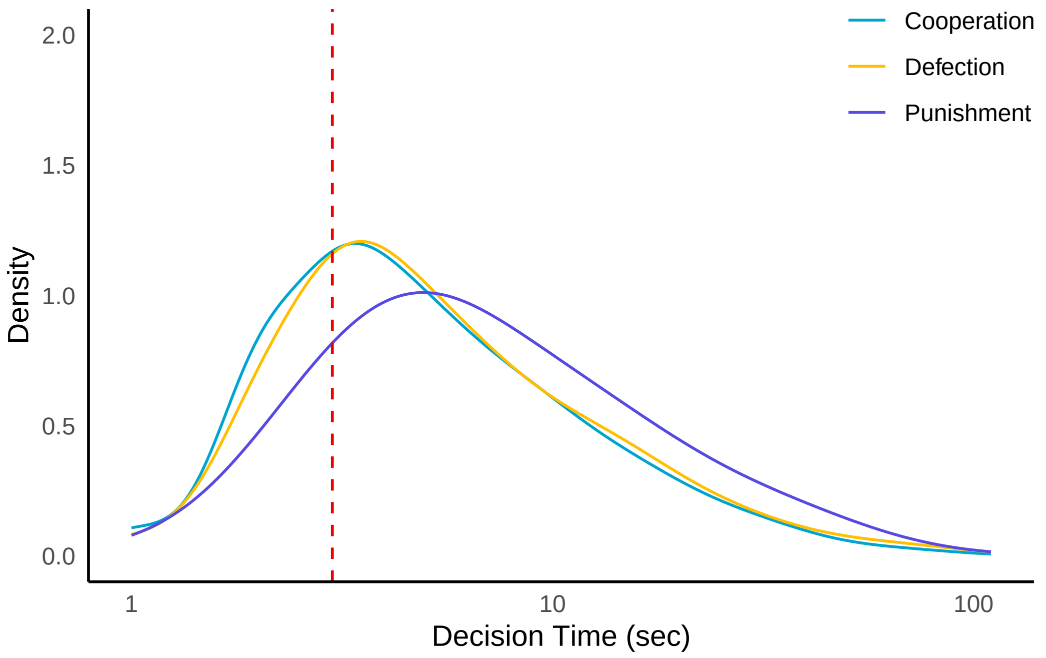


```
# ggsave(filename = "~/Documents/Projects/harming_esn/figures/figS2.png",
# width = 7, height = 5, units = "in")
```

Figure S3 - Sensitivity Analysis for Invisible Wealth Games Only (Exp. 1)

```
exp1data_invis_only = exp1data %>% filter(showScore == 0)

exp1data_invis_only_NR = exp1data_invis_only %>%
  group_by(punish_type_NR) %>%
  count() %>%
  ungroup() %>%
  mutate(punish_type = "NR",
         total = sum(n),
         perc = n/sum(n),
         se_perc = sqrt((perc*(1-perc))/total)) %>%
```

```
  filter(punish_type_NR == 1)  %>%
  select(punish_type, n, total, perc, se_perc)

exp1data_invis_only_IA = exp1data_invis_only %>%
  group_by(punish_type_IA) %>%
  count() %>%
  ungroup() %>%
  mutate(punish_type = "IA",
         total = sum(n),
         perc = n/sum(n),
         se_perc = sqrt((perc*(1-perc))/total)) %>%
  filter(punish_type_IA == 1)  %>%
  select(punish_type, n, total, perc, se_perc)

exp1data_invis_only_CR = exp1data_invis_only %>%
  group_by(punish_type_CR) %>%
  count() %>%
  ungroup() %>%
  mutate(punish_type = "CR",
         total = sum(n),
         perc = n/sum(n),
         se_perc = sqrt((perc*(1-perc))/total)) %>%
  filter(punish_type_CR == 1)  %>%
  select(punish_type, n, total, perc, se_perc)

exp1data_invis_only_U = exp1data_invis_only %>%
  group_by(punish_type_U) %>%
  count() %>%
  ungroup() %>%
  mutate(punish_type = "U",
         total = sum(n),
         perc = n/sum(n),
         se_perc = sqrt((perc*(1-perc))/total)) %>%
  filter(punish_type_U == 1)  %>%
  select(punish_type, n, total, perc, se_perc)

figS1data_invis_only =
  bind_rows(exp1data_invis_only_CR, exp1data_invis_only_IA,
            exp1data_invis_only_NR, exp1data_invis_only_U)

figS1data_invis_only = figS1data_invis_only %>%
  mutate(perc_LL = perc - 1.96*se_perc,
```

```
        perc_UL = perc + 1.96*se_perc)

exp1data_invis_only_NR_times = exp1data_invis_only %>%
  filter(punish_type_NR == 1, behavior_punish == 1) %>%
  summarize(mean_dt = mean1(behaviorTime_sec),
            se_mean_dt = se_mean(behaviorTime_sec)) %>%
  mutate(punish_type = "NR",
         mean_LL = mean_dt - 1.96*se_mean_dt,
         mean_UL = mean_dt + 1.96*se_mean_dt)

exp1data_invis_only_IA_times = exp1data_invis_only %>%
  filter(punish_type_IA == 1, behavior_punish == 1) %>%
  summarize(mean_dt = mean1(behaviorTime_sec),
            se_mean_dt = se_mean(behaviorTime_sec),
) %>%
  mutate(punish_type = "IA",   mean_LL = mean_dt - 1.96*se_mean_dt,
         mean_UL = mean_dt + 1.96*se_mean_dt)

exp1data_invis_only_CR_times = exp1data_invis_only %>%
  filter(punish_type_CR == 1, behavior_punish == 1) %>%
  summarize(mean_dt = mean1(behaviorTime_sec),
            se_mean_dt = se_mean(behaviorTime_sec)) %>%
  mutate(punish_type = "CR",
           mean_LL = mean_dt - 1.96*se_mean_dt,
         mean_UL = mean_dt + 1.96*se_mean_dt)

exp1data_invis_only_U_times = exp1data_invis_only %>%
  filter(punish_type_U == 1, behavior_punish == 1) %>%
  summarize(mean_dt = mean1(behaviorTime_sec),
            se_mean_dt = se_mean(behaviorTime_sec)) %>%
  mutate(punish_type = "U",
         mean_LL = mean_dt - 1.96*se_mean_dt,
         mean_UL = mean_dt + 1.96*se_mean_dt)

exp1data_invis_only_punish_types_times = bind_rows(exp1data_invis_only_NR_times,
                                        exp1data_invis_only_CR_times,
                                        exp1data_invis_only_IA_times,
                                        exp1data_invis_only_U_times) %>%
  select(punish_type, mean_dt, se_mean_dt, mean_LL, mean_UL)

fig2_data_invis_only = figS1data_invis_only %>%
  left_join(exp1data_invis_only_punish_types_times, by = "punish_type") %>%
```

```
  mutate(punish_type = case_match(punish_type,
             "CR" ~ "Copying/retaliation",
             "IA" ~ "Inequality aversion",
             "NR" ~ "Negative reinforcement",
             "U" ~ "Unclassified")) %>%
  mutate(punish_type_fct = factor(punish_type,
                                levels = c("Copying/retaliation",
                                           "Negative reinforcement",
                                           "Inequality aversion",
                                           "Unclassified")))
figS3_A = fig2_data_invis_only %>%
  ggplot(aes(x = punish_type_fct, y = perc, fill = punish_type_fct)) +
  geom_bar(position = "dodge", stat = "identity", alpha = 0.5, show.legend = F) +
  geom_errorbar(aes(ymin = perc + 1.96*se_perc,
                  ymax = perc - 1.96*se_perc,
                  width = 0.25),
              color = "purple4",
              position = position_dodge(.9),
              show.legend = F) +
  scale_fill_manual(values = c("Copying/retaliation" = "plum3",
                              "Negative reinforcement" = "mediumslateblue",
                              "Inequality aversion" = "mediumpurple3",
                              "Unclassified" = "orchid4"), guide = "none") +
  scale_color_manual(guide = "none") +
  scale_x_discrete(labels = c("Copying/\nretaliation", "Negative\nreinforcement",
                             "Inequality\naversion", "Unclassified")) +
  scale_y_continuous(limits = c(0, 0.05), expand = c(0, 0)) +
  ylab("Proportion") +
  xlab("") +
  labs(tag = "A") +
  theme_classic() +
  theme(panel.grid.minor = element_blank(),
        panel.grid.major = element_blank(),
        legend.position = "bottom",
        legend.title = element_blank(),
        axis.text.x = element_text(size = 8),
        axis.title.x = element_blank(),
        axis.ticks.x = element_blank(),
        axis.ticks.y = element_blank())
```

```
figS3_B = fig2_data_invis_only %>%
  ggplot(aes(x = punish_type_fct, y = mean_dt, fill = punish_type_fct)) +
```

```
  geom_bar(stat = "identity", width = 0.75,  alpha = 0.5) +
  geom_errorbar(aes(ymin = mean_LL,
                    ymax = mean_UL),
                color = "purple4", width = 0.25) +
  theme_classic() +
  scale_y_continuous(limits = c(0, 12), breaks = seq(0, 12, by = 2), expand = c(0, 0)) +
  ylab("Decision time \n (seconds)")+
  scale_fill_manual(values = c("Copying/retaliation" = "plum3",
                               "Negative reinforcement" = "mediumslateblue",
                               "Inequality aversion" = "mediumpurple3",
                               "Unclassified" = "orchid4"), guide = "none") +
  scale_x_discrete(labels = c("Copying/\nretaliation", "Negative\nreinforcement",
                              "Inequality\naversion", "Unclassified")) +
  scale_color_manual(guide = "none") +
  theme_classic() +
  labs(tag = "B") +
  theme(panel.grid.minor = element_blank(),
        panel.grid.major = element_blank(),
        legend.position = "none",
        axis.text.x = element_text(size = 8),
        axis.title.x = element_blank(),
        axis.ticks.x = element_blank(),
        axis.ticks.y = element_blank())
```

```
figS3_A + figS3_B
```

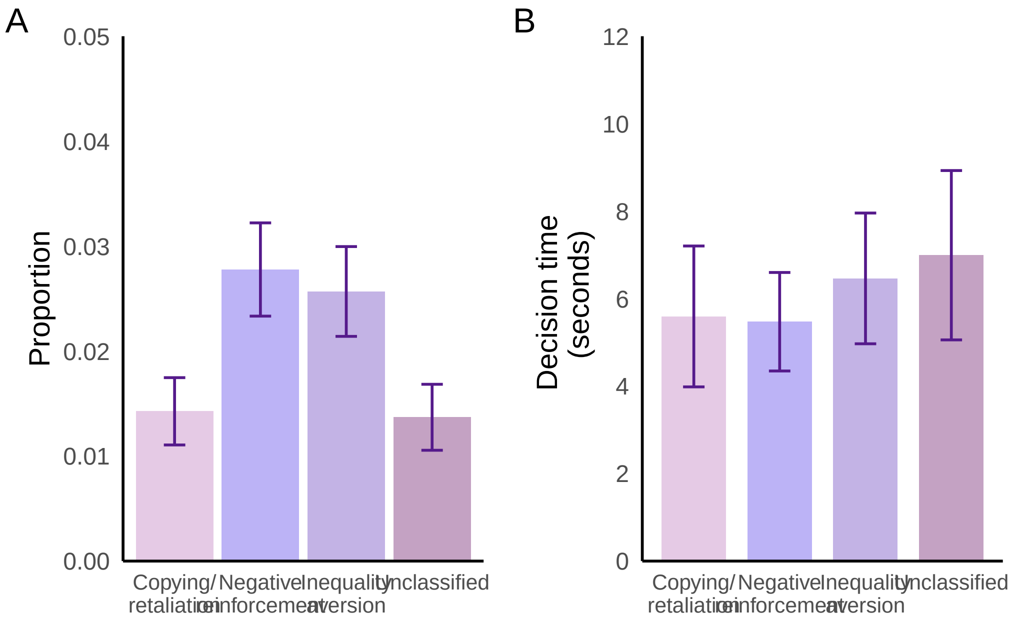


```
# ggsave(filename = "~/Documents/Projects/harming_esn/figures/figS3.png",
#        width = 8, height = 5, units = "in")
```
